# Cathelicidin peptide analogues inhibit EV71 infection through blocking viral entry and uncoating

**DOI:** 10.1371/journal.ppat.1011967

**Published:** 2024-01-25

**Authors:** Tingting Fan, Bing Liu, Haoyan Yao, Xinrui Chen, Hang Yang, Shangrui Guo, Bo Wu, Xiaozhen Li, Xinyu Li, Meng Xun, Hongliang Wang

**Affiliations:** 1 Department of Infectious Diseases, The First Affiliated Hospital of Xi’an Jiaotong University, Shaanxi, China; 2 Department of Pathogen Biology and Immunology, Xi’an Jiaotong University Health Science Center, Shaanxi, China; 3 Biobank, The First Affiliated Hospital of Xi’an Jiaotong University, Shaanxi, China; 4 Department of Gynecology and Obstetrics, The First Affiliated Hospital of Xi’an Jiaotong University, Shaanxi, China; National University of Singapore Yong Loo Lin School of Medicine, SINGAPORE

## Abstract

Given the serious neurological complications and deaths associated with enterovirus 71 (EV71) infection, there is an urgent need to develop effective antivirals against this viral infection. In this study, we demonstrated that two Cathelicidin-derived peptides, LL-18 and FF-18 were more potent against EV71 infection than the parent peptide LL-37, which is the mature and processed form of Cathelicidin. These peptides could directly bind to the EV71 virus particles, but not to coxsackievirus, indicative of their high specificity. The binding of peptides with the virus surface occupied the viral canyon region in a way that could block virus-receptor interactions and inhibit viral uncoating. In addition, these peptide analogues could also relieve the deleterious effect of EV71 infection *in vivo*. Therefore, Cathelicidin-derived peptides might be excellent candidates for further development of antivirals to treat EV71 infection.

## Author summary

Enterovirus 71(EV71) is one of the major pathogens that cause hand, foot, and mouth disease, and in some cases severe neurological symptoms. There is currently no approved antiviral for EV71. Here in this study, we demonstrated that two antimicrobial peptides derived from the Cathelicidin family, namely, LL-18 and FF-18 were effective against EV71 infection *in vitro* and *in vivo*. These peptides could bind directly to the viral particle surface and prevent the virus from entering the host cell as well as blocking successful viral genome release. In addition, we also showed that LL-18 has a low propensity to induce virus resistance. Therefore, these peptides represent promising candidates for EV71 antivirals.

## Introduction

Hand, foot, and mouth disease (HFMD) caused by enteroviruses is a serious public health threat, especially in the Asia-Pacific region [1]. Enterovirus 71 (EV71) and coxsackievirus A16 (CVA16) are the main pathogens of HFMD, which usually infect children under 6 years of age. In particular, due to its high neurotropism feature, EV71 infection is usually associated with severe neurological complications or even death [2]. Due to the lack of effective antivirals for treatment, supportive therapy remains to be the primary measure for severe infections.

EV71 belongs to the genus *Enterovirus* in the family of *Picornavirridae*. Its genome is a single-stranded, positive sense RNA of ~7400 nucleotides long [3]. The viral genome encodes four structural proteins VP1-VP4, which assemble to form a protomer with VP1-VP3 exposed on the capsid surface, while VP4 is attached to the inner surface. Five such protomers constitute a pentamer and 12 pentamers together form a virion, which encloses the viral genome [4]. Crystal structure showed that a depression (the “canyon”) encircles the icosahedral fivefold axes of the virions, which was predicted to be the binding site of receptors that have an immunoglobulin-like fold [5]. Several molecules have been found to act as receptors for EV71 infection, including Scavenger receptor class B member 2 (SCARB2), P-selectin glycoprotein ligand-1(PSGL-1), annexin II, heparan sulfate (HS), etc [6,7]. Among them, SCARB2 is the major function receptor that supports both attachment and uncoating of virions, while others are classified as “attachment receptors” [6,7]. In addition, a non-protein molecule, termed “pocket factor” was found to reside in the hydrophobic pocket underneath the canyon in most enteroviruses, which stabilizes the capsid [5,8,9]. Receptor binding into the canyon causes conformational changes that expulse the pocket factors from the hydrophobic pockets and initiate the uncoating process [4,10,11].

Antimicrobial peptides (AMP), which are important effectors of the innate immune system, are critical defense lines against various pathogen infections in humans. The Defensin and Cathelicidin family peptides are two major classes of AMP produced in the human body [12]. The Defensin group consists of a variety of antimicrobial peptides, while human cationic antimicrobial protein (hCAP18) is the only member of the Cathelicidin family identified in human cells [12]. hCAP18 mainly expresses in bone marrow cells and epithelial cells of many organs, and its C-terminal bioactive domain, LL-37, which shows various effects, such as antibacterial, wound healing promotion, and immune regulation, is generated through proteolytic cleavage [13]. LL-37 has also been shown to inhibit viral infections, including influenza, HIV, respiratory syncytial virus (RSV), and Dengue virus, most of which are enveloped viruses [14,15]. In addition to the native peptide, modified or designed peptides have also been shown to have antimicrobial or antitumor effects [12]. For example, LL-18 and FF-18, which are 27-residue peptide analogues of LL-37, have been shown to possess antibacterial effects against bacteria strains belonging to the genera *Porophyromonas* and *Prevotella* [16].

In this study, we found that the Cathelicidin peptide analogues, LL-18 and FF-18 showed more potent antiviral effects against EV71 infection, but could not inhibit echovirus or coxsackievirus infection. Further mechanism studies showed that these peptides could directly bind to the EV71 virus particles, occupying the viral canyon region and blocking virus-receptor interactions. Moreover, through this direct interaction, they also inhibited viral uncoating, thus exhibiting their effective antiviral effects. Finally, we showed that LL-18 could also relieve the deleterious effect of EV71 infection *in vivo*, suggesting these peptides could be developed into novel antivirals to treat EV71 infection.

## Results

### LL-18 and FF-18 effectively inhibit EV71 infection

**Fig 1:**
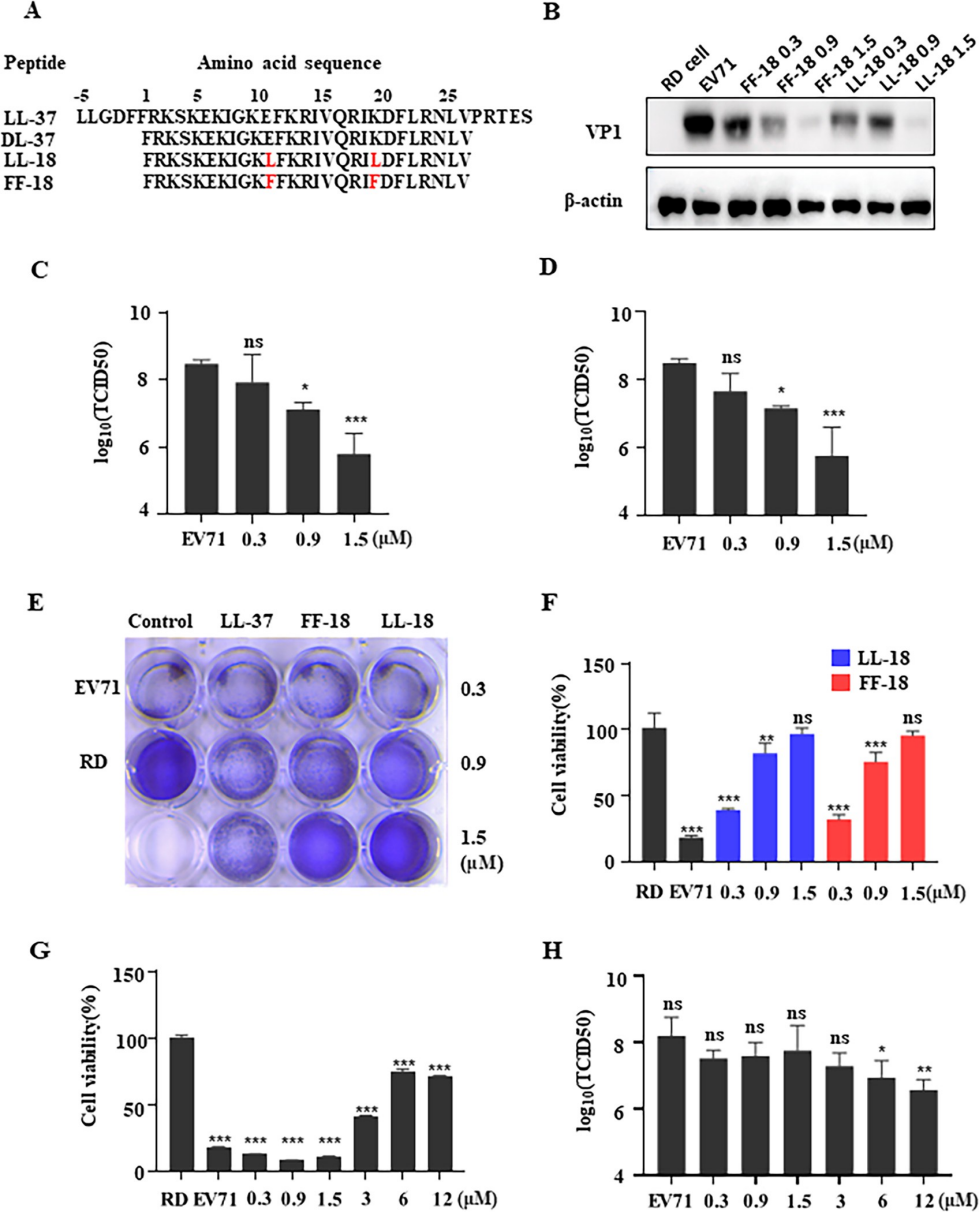
LL-18 and FF-18 effectively inhibit EV71 infection. (A) The amino acid sequences of the peptides used. The numbers above indicate the amino acid residues. For better comparison, the first “F” in DL-37 was designated as the 1^st^ residue. (B) EV71 virus (MOI = 1) pre-incubated with indicated amounts of peptides were used to infect RD cells and viral VP1 expression was determined with immunoblotting. β-actin was used as loading control. (C-D) EV71 virus pre-incubated with indicated amounts of LL-18 (C) or FF-18 (D) and the viral titer in the supernatant was determined 24 h.p.i. ns, not significant; *, P<0.05; ***, P<0.001. (E-F) EV71 virus pre-incubated with indicated amounts of peptides were used to infect RD cells. Cells were then fixed and stained with crystal violet (E) or assayed for viability with Cell Titer Glo (F) 24 hrs post-infection (h.p.i). ns, not significant; **, P<0.01; ***, P<0.001. (G-H) EV71 virus pre-incubated with indicated amounts of LL-37 and cell viability (G) or viral titer (H) was determined 24 h.p.i. Values are normalized to uninfected RD cells. ns, not significant; *, P<0.05; **, P<0.01; ***, P<0.001.

To test whether Cathelicidin peptide (LL-37) and its analogues could inhibit enterovirus infection, we conducted viral infection inhibition test on RD cells, which are one of the most commonly used cells for enterovirus propagation. We first assessed the cytotoxicity of LL-37, LL-18, FF-18, and a short form of LL-37 (DL-37) (Fig 1A), and all the peptides tested have a CC_50_ over 50 μM (S1A–S1D Fig). We then chose lower concentrations to test their antiviral activity. LL-18 and FF-18 were shown to inhibit EV71 viral protein expression in a dose-dependent manner (Fig 1B). Consistent with this, viral titration showed that LL-18 and FF-18 could also reduce the viral titers dose-dependently (Fig 1C and 1D). In addition, EV71 infection could cause cytopathic effect (CPE) on RD cells, and peptide could rescue EV71-induced cell death as assayed by crystal violet staining (Fig 1E) or cellular ATP content measurement (Fig 1F). All these results suggest that LL-18 and FF-18 could effectively inhibit EV71 infection. In contrast, LL-37, or DL-37 could not inhibit EV71 infection at 0.3, 0.9, or 1.5 μM and only showed inhibition at a much higher concentration (Figs 1G, 1H and S1E), suggesting Cathelicidin analogue LL-18 or FF-18 is much more potent against EV71 infection.

We then tested whether these peptides could also inhibit coxsackievirus B5 (CVB5) or Echovirus 7 (Echo7) infection, both of which are close relatives of EV71. At concentrations that could effectively inhibit EV71 infection, neither LL-18 nor FF-18 could inhibit CVB5 or Echo 7 infection (S1F and S1G Fig). When higher concentrations were tested, we found LL-18 or FF-18 could still not inhibit CVB5 or Echo 7 infection (S1H and S1I Fig). These results together indicate that LL-18 or FF-18 could specifically inhibit EV71 infection.

### LL-18 and FF-18 inhibit EV71 infection at the early stages of infection

**Fig 2:**
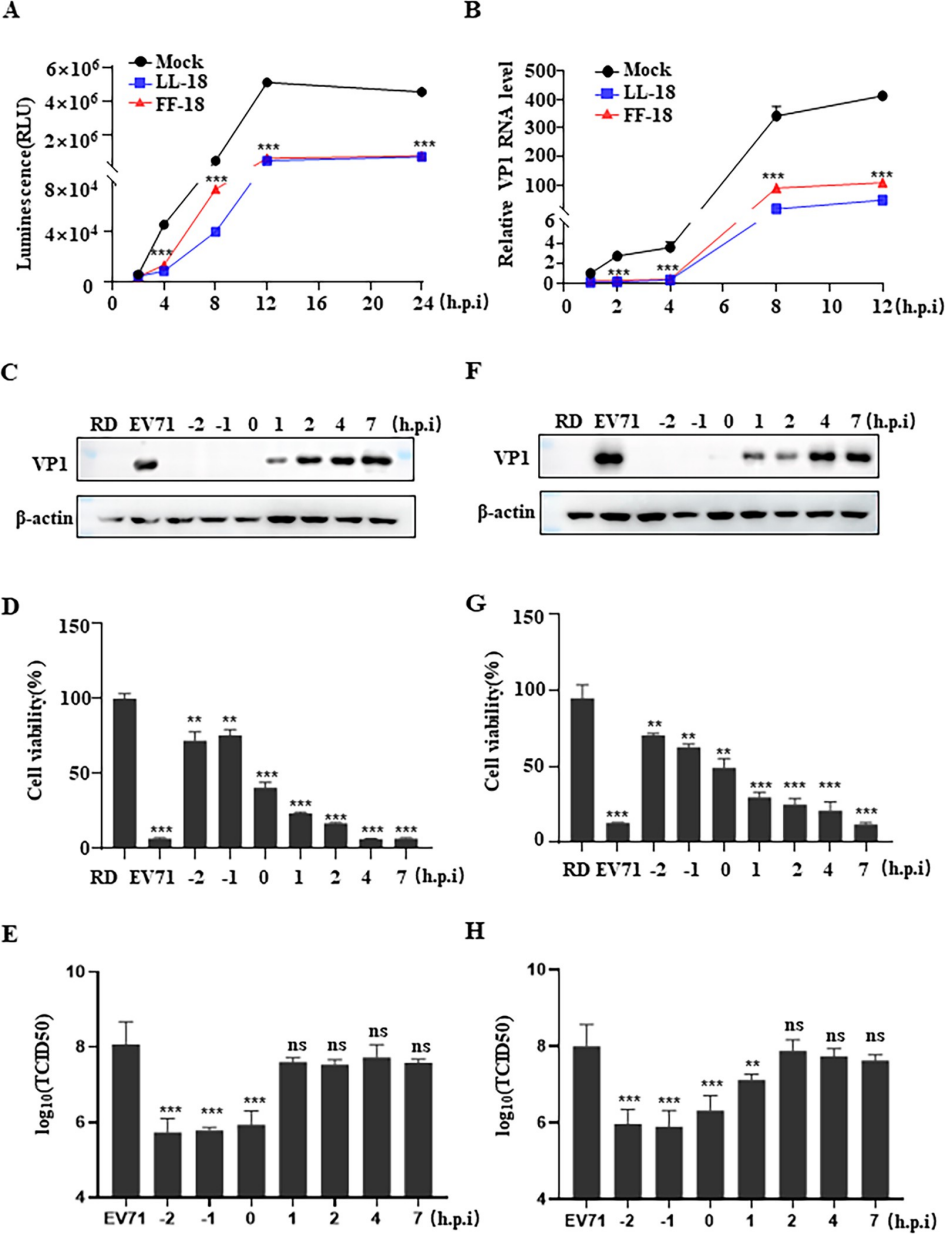
LL-18 and FF-18 inhibit EV71 infection at the early stages of infection. (A-B) NanoLuc-EV71 reporter virus (A) or EV71 virus (MOI = 1, B) was pre-incubated with 1.5 μM LL-18 or FF-18 for 1 hr before they were used to infect RD cells. Luciferase (A) or viral RNA (B) was determined at indicated time points. Viral RNAs were normalized to mock treated cells at 1 h.p.i. ***, P<0.001. (C-E) EV71 virus (MOI = 1) was either pre-incubated with 1.5 μM LL-18 before infection (-2 and -1 time points), added simultaneously with LL-18 (0 time point) or LL-18 was added at indicated time points post-infection (1, 2, 4, 7 time points) and viral VP1 expression (C), cell viability (D), or viral titer (E) was determined 24 h.p.i. ns, not significant; **, P<0.01; ***, P<0.001. (F-H) EV71 virus (MOI = 1) was either pre-incubated with 1.5 μM FF-18 before infection (-2 and -1 time points), added simultaneously with FF-18 (0 time point) or FF-18 was added at indicated time points post-infection (1, 2, 4, 7 time points) and viral VP1 expression (F), cell viability (G), or viral titer (H) was determined 24 h.p.i. ns, not significant; **, P<0.01; ***, P<0.001.

To gain insights on how LL-18 and FF-18 inhibit EV71 infection, we next aimed to find out at which stage LL-18 or FF-18 blocks EV71 infection. To this aim, we infected RD cells with the NanoLuc-EV71 reporter virus. A NanoLuc reporter gene was inserted upstream of the viral major ORF and the modified genome could still be encapsidated into infectious viral particles, and therefore, it can be used to study viral infection by monitoring the bioluminescence. [17] Luciferase activity at various time points was monitored and we found that LL-18 or FF-18 could inhibit the expression of NanoLuc as early as 4 hrs post-infection (h.p.i) (Fig 2A). Similarly, when cells were infected with parental EV71 virus that has been pre-incubated with peptide, viral RNA was observed to be reduced as early as 2 h.p.i (Fig 2B). These results suggested that these peptides block EV71 infection at the early stage of infection.

To further find out the time point when these peptides function, we carried out a time-of-addition study, during which peptide analogues were added at various time points prior to or post the addition of the EV71 virus. Potent inhibition of viral protein expression (Fig 2C), virus-induced cell death (Fig 2D), or virus titer (Fig 2E) was observed when LL-18 was added before virus infection, while it had little inhibition when added after virus infection. Similar results were obtained when FF-18 was tested (Fig 2F, 2G and 2H). These results suggested that LL-18 or FF-18 blocks EV71 infection at the early stage of infection, most likely during the entry process.

### LL-18 and FF-18 inhibit viral attachment and internalization

**Fig 3:**
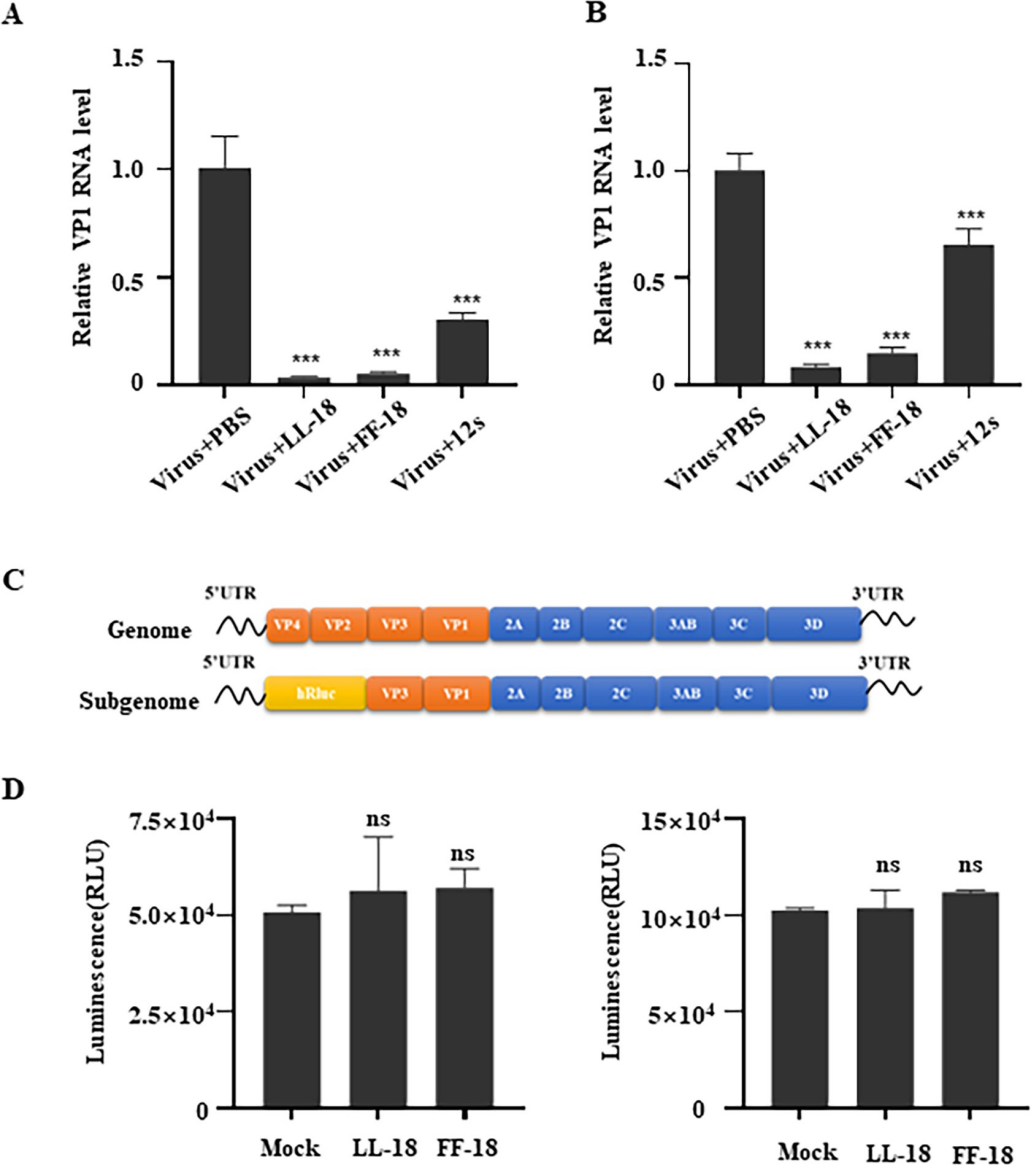
LL-18 and FF-18 inhibit viral attachment and internalization. (A) EV71 virus (MOI = 1) was pre-incubated with 1.5 μM LL-18, FF-18 or 12 μM 12s before they were used to incubate with RD cells at 4°C for 1 hr. Viral RNA was then determined with q-RT-PCR. Values are normalized to vehicle treated cells. ***, P<0.001. (B) EV71 virus (MOI = 1) was pre-incubated with 1.5 μM LL-18, FF-18 or 12 μM 12s before they were used to incubate with RD cells at 4°C for 1 hr followed by 37°C incubation for 1 hr. Viral RNA was then determined with q-RT-PCR. Values are normalized to vehicle treated cells. ***, P<0.001. (C) Diagram of EV71 subgenomic replicon. Part of the structural genes was replaced with a *Renilla* luciferase reporter gene. (D) RD cells transfected with EV71 SGR RNA were treated with 1.5 μM LL-18 or FF-18 for 3 hrs (left panel) or 6 hrs (right panel) before luciferase activity was measured. ns, not significant.

We next explored whether LL-18 or FF-18 affects viral attachment and internalization. The aminothiazole derivative (12**s**), a small molecule compound reported to inhibit EV71 by interfering with viral VP1-SCARB2 interaction [18], was used as a positive control. EV71 viruses were pre-incubated with PBS, 12s, LL-18, or FF-18 and then incubated with RD cells at 4°C for 1 hr to allow virus attachment to host cells. Both LL-18 and FF-18 could effectively inhibit EV71 attachment when attached viruses were quantified with q-RT-PCR (Fig 3A) or subjected to immunoblotting (S2A Fig). When attached viruses were allowed to internalize at 37°C for 1 hr, strong inhibition was also observed for LL-18 or FF-18 treatment (Figs 3B and S2B). These results suggest that LL-18 and FF-18 inhibit viral attachment and internalization.

To test whether LL-18 or FF-18 inhibits viral translation or replication, we employed the subgenomic replicon (SGR) of EV71, in which part of the structural gene was replaced with a luciferase reporter gene (Fig 3C) and could not package into new virions [17]. The SGR is *in vitro* transcribed and transfected into RD cells and thus bypasses the viral entry steps. SGR-transfected cells were treated with LL-18 or FF-18 for 3 or 6 hrs, and no inhibition was observed for either treatment (Fig 3D), suggesting LL-18 or FF-18 could not affect viral translation or replication. Overall, these results suggested that LL-18 or FF-18 inhibited viral entry into host cells but could not inhibit viral genome replication.

### LL-18 binds directly to the virus

Now that we know LL-18 and FF-18 could block EV71 entry, we next sought to know whether LL-18 and FF-18 could bind to virus or host cells to block the interaction between virus and host cells. As shown above, if we pre-incubate the virus with LL-18 or FF-18, they would block viral infection. However, if we incubated cells with peptides first for 2 hrs and then washed out the unbound peptides, we found EV71 could still infect these treated cells and cause cell death (S3A Fig). These results indicate that LL-18 and FF-18 most likely bind to viruses to block their entry.

**Fig 4:**
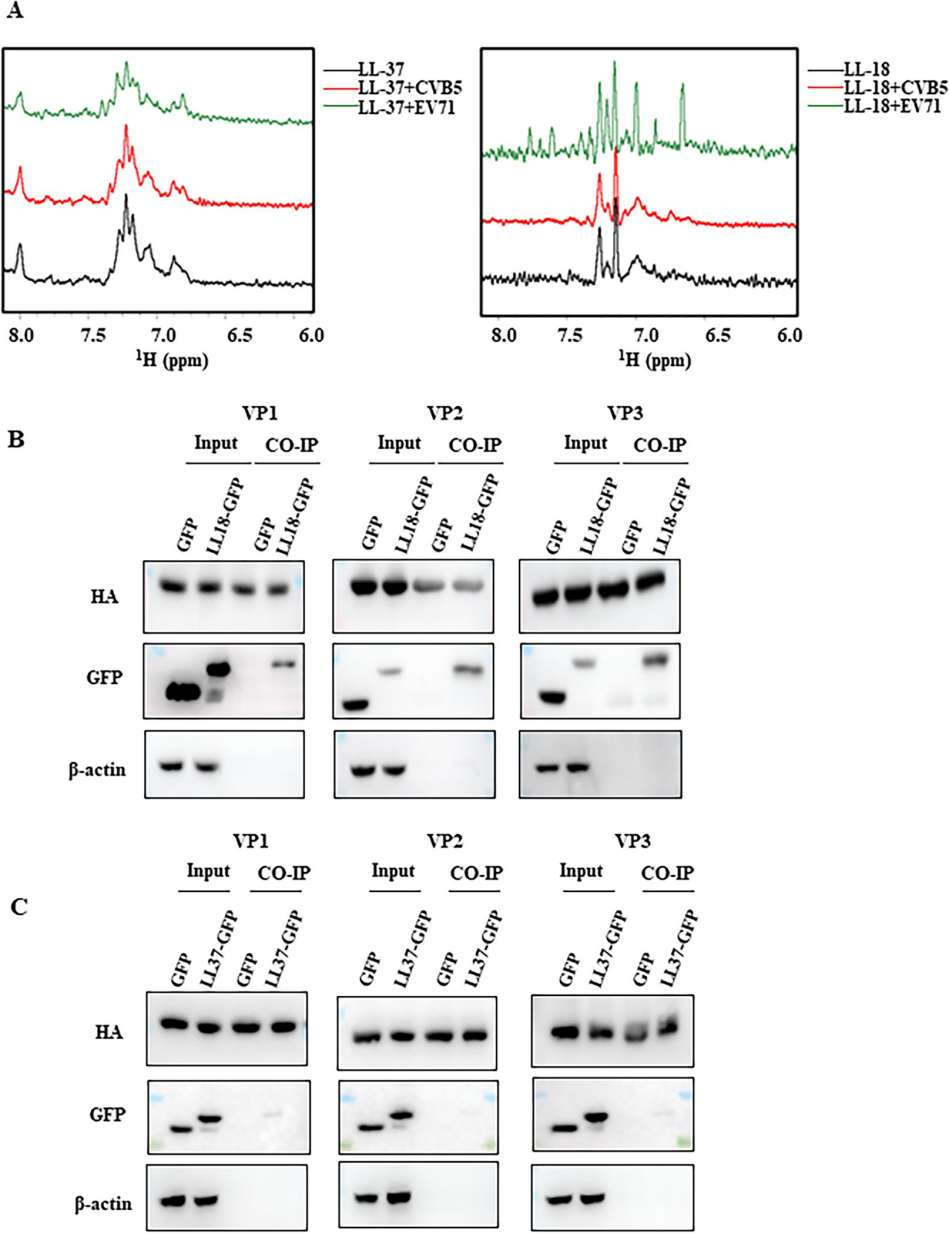
LL-18 binds directly to virus particles. (A) ^1^H NMR data showing the chemical shifts. Spectra of LL-37 (left panel) or LL-18 (right panel) peptide in the absence or presence of EV71 virus (green line) or CVB5 virus (red line). (B-C) 293T cells cotransfected with GFP or GFP-tagged LL-18 (B) or LL-37 (C) and HA-tagged VP1, VP2, or VP3 plasmids were immunoprecipitated with anti-HA antibody, and interactions between LL-18 and viral structural proteins were detected by anti-GFP antibody.

To verify the possible interactions between the peptides and the viruses, we employed nuclear magnetic resonance (NMR) titration and examined the changes in the amide region of the peptide ^1^H 1D spectra before and after adding the relevant virus. In the LL-37, adding either EV71 or CVB5 had no effect on the peak intensity or the chemical shift of the peaks, suggesting no interaction between the peptide and viruses (Fig 4A left panel). In contrast, when EV71 virus was added to LL-18 peptide, chemical shift perturbation with multiple new peaks was observed, indicating the interactions between LL -18 and EV71; while the negative control virus, CVB5 had little effect on the spectra of peptides (Fig 4A right panel). Similar results were obtained when EV71 or CVB5 virus was added in FF-18 peptide (S3B Fig). These results confirmed direct interactions between EV71 virus and LL-18 or FF-18 peptides.

Since capsid proteins VP1-VP3 are located on the outside of the capsid, we next tested whether LL-18 could interact with these viral capsid proteins with immunoprecipitation. While LL18-GFP coimmunoprecipitated with VP1, VP2, and VP3, little interaction was observed with LL37-GFP and viral VP1, VP2 or VP3 (Fig 4B and 4C). These results suggested that LL-18 could interact with the outside of the EV71 capsid.

### LL-18 interferes with virus-SCARB2 interaction

The fact that LL-18 could interact with all three outside capsid proteins indicates that this peptide might bind to a site where VP1, VP2, and VP3 associate together on the surface of virions. Interestingly, the “canyon” was formed by VP1, VP2, and VP3 proteins, and SCARB2 binds EV71 on the southern rim of the canyon and interacts directly with VP1 and VP2 loops [19]. We then speculated that LL-18 could interact with EV71 around the canyon region, which blocks its interaction with SCARB2 and inhibits entry.

**Fig 5:**
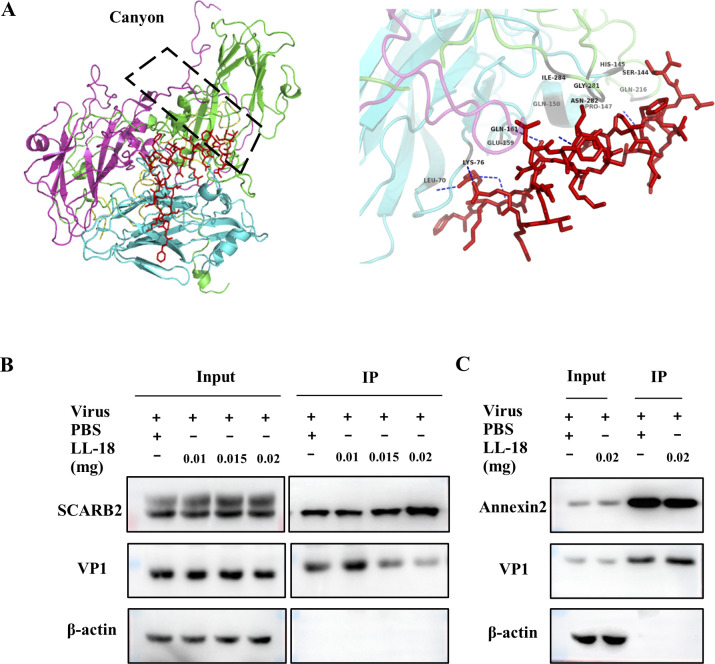
LL-18 interferes with virus-SCARB2 interaction. (A) Molecular docking model of LL-18 with EV71 virion. EV71 capsid proteins VP1 (green), VP2 (pink), and VP3(blue), all interact with LL-18 displayed in red sticks (left panel). The amino acid residues of capsid protein interacting with both LL-18 and SCARB2 are indicated (right panel). (B) 293T cells expressing SCARB2-Flag were lysed and cell lysates were incubated with EV71 virus in the absence or presence of the indicated amount of LL-18. Cell lysates were then immunoprecipitated with anti-Flag antibody and the precipitated virus was determined with anti-VP1 antibody. (C) 293T cells expressing Annexin 2-Flag were lysed and cell lysates were incubated with EV71 virus in the absence or presence of LL-18. Cell lysates were then immunoprecipitated with anti-Flag antibody and the precipitated virus was determined with anti-VP1 antibody.

A docking simulation model showed that LL-18 binds to sites in proximity to the “canyon” site where VP1, VP2, and VP3 of EV71 meet together (Fig 5A). The specific interaction residues are shown in S1 Table, among which residues Gln216, Gly281, Asn282, and Ile284 of VP1, as well as residues Ser144, His145, Pro147, and Gln150 of VP2, have been shown to mediate the binding of EV71 to SCARB2 [20]. To confirm whether the presence of LL-18 interferes with virus-receptor interaction, we carried out the following experiment, in which we incubated the virus with SCARB2 in the absence or presence of increasing amounts of LL-18 or 12s. The amount of viral particles that were coimmunoprecipitated with SCARB2 was measured with immunoblotting with an antibody against VP1. As 12s has been reported to interfere with VP1-SCARB2 interaction, less VP1 was coimmunoprecipitated in the presence of 12s (S4 Fig). Similarly, LL-18 could also inhibit virus binding to SCARB2 in a dose-dependent manner (Fig 5B). In contrast, Annexin 2, another receptor for EV71 with a distinct binding site on virion [21], was not competed out by LL-18 (Fig 5C). These results suggest that LL-18 interacts with EV71 viral particles that block its binding sites to its receptor-SCARB2.

### LL-18 stabilizes the viral particles by inhibiting uncoating

**Fig 6:**
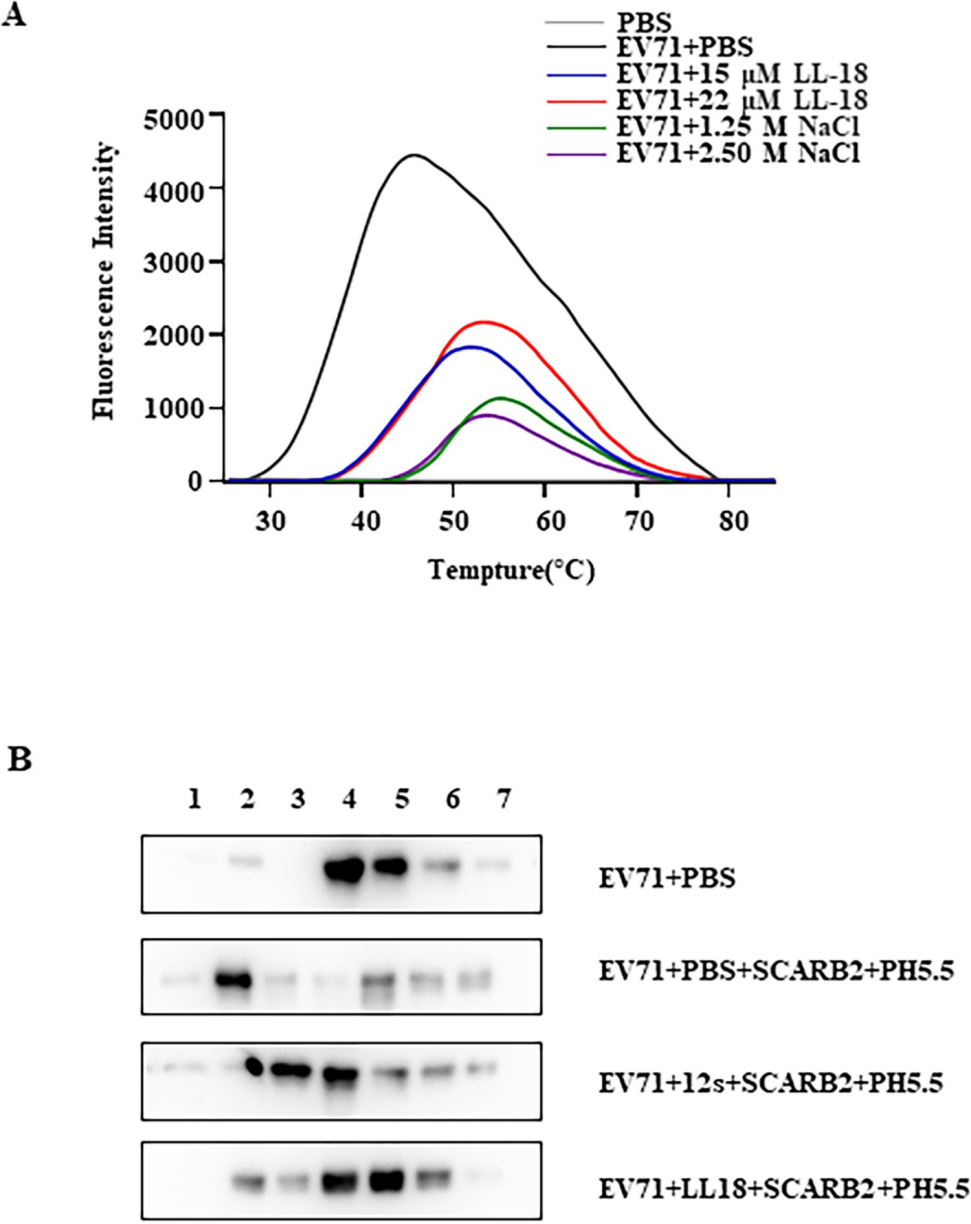
LL-18 stabilizes the viral particles by inhibiting uncoating. (A) EV71 virus (MOI = 10) was pre-incubated with PBS, indicated amount of LL-18 or NaCl for 1 hr and then mixed with SYBR green dyes II before they were heated to indicated temperatures. The fluorescent signals were measured and plotted against temperature. PBS was used as a negative control. (B) EV71 virus (MOI = 10) was pre-incubated with PBS, LL-18, or 12s for 2 hrs. Half of the samples were then treated with 1.5 μg SCARB2 protein and incubated at 37°C for 2 hrs at pH 5.5. Samples were then subjected to ultracentrifugation as detailed in materials and methods. The numbers above represent the fractions collected from top to bottom.

Receptor binding to the canyon region will lead to the release of the “pocket factor” and trigger the uncoating process, while small molecular inhibitors binding to the hydrophobic pocket region with high affinity would block viral uncoating upon receptor binding [22,23]. Since LL-18 binds to the viral surface in proximity to the “canyon” region, we speculate it might also affect viral uncoating. To test this hypothesis, we carried out the viral particle stability thermal release assay (PaSTRY) [24] in the presence of LL-18 or high concentration of NaCl, which has been shown to increase virus thermostability by enhancing the van der Walls forces at the protein interfaces [25]. SYBR green could not penetrate into native virions due to the sealed protein shell, while viral uncoating would expose the viral genome and allow the accessibility of nucleic acid to SYBR dyes. Fig 6A showed that RNA genome in EV71 virions incubated with LL-18 was less accessible to SYBR green. The virus treated with PBS generated peak fluorescence intensity, indicating RNA release around 45°C, while in the presence of NaCl, the fluorescence intensity peak shifted to 55°C. Similarly, LL-18 could also shift the fluorescence intensity peak to 53°C. These results indicate that LL-18 enhances viral stability by limiting RNA release.

To confirm this result, LL-18 treated viral particles were subjected to gradient centrifugation to discriminate native and disassembled virions. It has been reported that uncoating of EV71 occurred in a SCARB2-dependent, low pH environment [26,27]. Therefore, treatment of EV71 virions with PBS did not cause any uncoating, while virions treated with low pH in the presence of SCARB2 led to VP1 floated to lower density (Fig 6B upper panels), indicative of uncoating. However, in the presence of LL-18, or 12s, to a lesser extent, EV71 virions remained in the higher density fractions (Fig 6B lower panels), suggesting LL-18 inhibited the uncoating.

### LL-18 inhibits EV71 infection *in vivo*

To test the antiviral effect of LL-18 *in vivo*, we first established an EV71 mouse infection model. As reported, mouse-adaption could increase the virulence of EV71 in mice [28] and therefore, we adapted our strain with serial passages in mice. As expected, this adapted virus (mEV71) caused significant mortality in 6-day-old mice, while the parental EV71 could not kill the mice (S5A Fig). Importantly, LL-18 could still inhibit the infection of this mEV71 strain in RD cells (S5B Fig). To find out the optimal dose of peptide usage *in vivo*, we did a preliminary test by measuring the virus titers with different doses of LL-18 treatment. S5C Fig showed that 20 mg/kg LL-18 could significantly reduce the viral load and that dose was chosen for further analysis.

**Fig 7:**
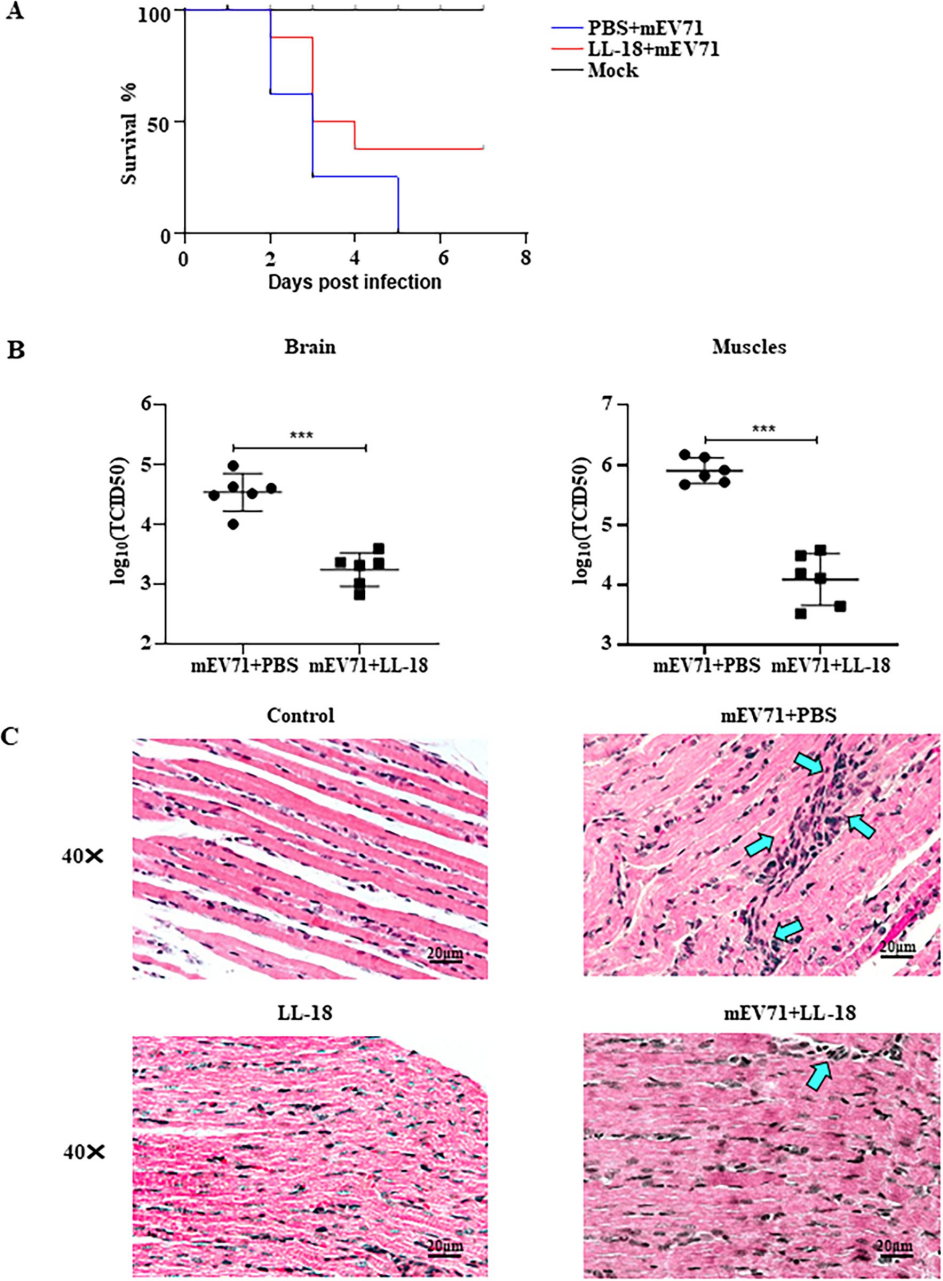
LL-18 inhibits mEV71 infection *in vivo*. (A) mEV71 was pre-treated with PBS or LL-18 and used to infect 6-day old ICR mice. The survival rate of mice infected with the mEV71 virus with or without LL-18 was recorded. (B) Mice infected with the mEV71 virus with or without LL-18 pre-treatment were sacrificed 1 day post infection (d.p.i.) and viral titers from brain and muscle tissues were determined. ***, P<0.001. (C) H&E staining of muscle tissue from mice infected with the mEV71 virus with or without LL-18. Blue arrows indicate infiltrated neutrophils. Bar, 20μm.

The mEV71 was then pre-incubated with an equal volume of PBS or LL-18 (20mg/kg) respectively for 1 hour, and the mixture was injected intraperitoneally into the 6-day-old ICR mice. Fig 7A showed that LL-18 could increase the sucking mouse survival rate. Meanwhile, the viral loads of brain and muscle from infected mice were also determined on 1 d.p.i, and Fig 7B showed that LL-18 significantly decreased the viral loads in both brain and muscle. Histological examination showed that mEV71 infection caused inflammatory cell infiltration (blue arrows), which was improved with the administration of LL-18 (Fig 7C). These results suggest that LL-18 could reduce EV71 infection *in vivo*.

### The 20^th^ amino acid residue is essential for the antiviral effect of LL-18

**Fig 8:**
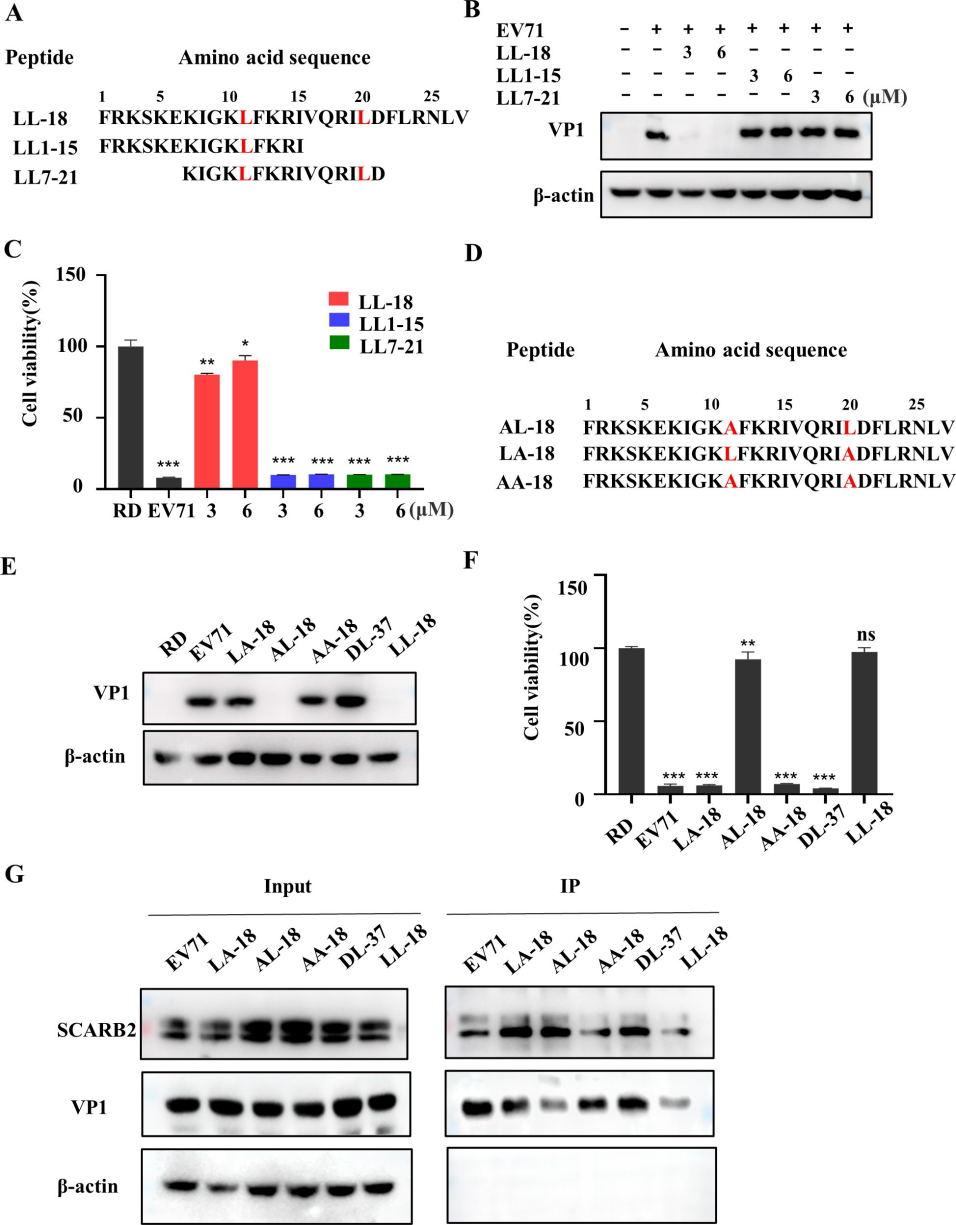
The 20^th^ residue of the peptide is essential for its antiviral activity. (A) The amino acid sequence of LL1-15 and LL7-21. (B-C) EV71 virus pre-incubated with indicated amounts of LL1-15 or LL7-21 was used to treat cells and viral VP1 expression (B) or cell viability (C) was determined 24 h.p.i. Values are normalized to uninfected RD cells. *, P<0.05; **, P<0.01; ***, P<0.001. (D) The amino acid sequence of AL-18, LA-18 and AA-18. (E-F) EV71 virus pre-incubated with 1.5 μM AL-18, LA-18 or AA-18 was used to treat cells and viral VP1 expression (E) or cell viability (F) was determined 24 h.p.i. Values are normalized to uninfected RD cells. ns, not significant; **, P<0.01; ***, P<0.001. (G) 293T cells expressing SCARB2-Flag were lysed and cell lysates were incubated with EV71 virus in the absence or presence of the indicated peptides. Cell lysates were then immunoprecipitated with anti-Flag antibody and the precipitated virus was determined with anti-VP1 antibody.

The peptide length is an important parameter that influences peptide binding and drug delivery [29, 30]. Now that we know 27 amino acid-long peptides are superior to 37 amino acids, we next wanted to know whether shortening the peptide will improve potency or bioavailability. For this purpose, we designed two shorter peptides: LL1-15 and LL7-21(Fig 8A). Notably, based on the structure of LL-37 and the docking model of LL-18 with EV71 virion, LL7-21 bears all the essential residues that interact with EV71 and docking predicts that it could still bind to EV71 (S6 Fig). However, neither peptide could inhibit viral VP1 expression (Fig 8B) or rescue EV71-induced cell death (Fig 8C), suggesting they lost their antiviral potency.

The fact that DL-37 has little antiviral activity, while the LL-18 or FF-18, both of which bear substituted 11^th^ and 20^th^ amino acid residues, have potent antiviral activity, prompted us to speculate that these two residues play important roles in their antiviral activity. We therefore designed peptides of AL-18, LA-18, and AA-18, which had alanine substitution at the 11^th^, 20^th^ residue or both (Fig 8D). Fig 8E and 8F showed that AL-18 could still exhibit antiviral effect, but LA-18 or AA-18 lost their ability to inhibit viral infection. Consistent with this, AL-18 could inhibit virus interaction with SCARB2, while LA-18 or AA-18 could not (Fig 8G). These results suggest that the 20^th^ residue of LL-18 was the most important residue for its antiviral activity and virus interaction.

### LL-18 has a low propensity to induce virus resistance

**Fig 9:**
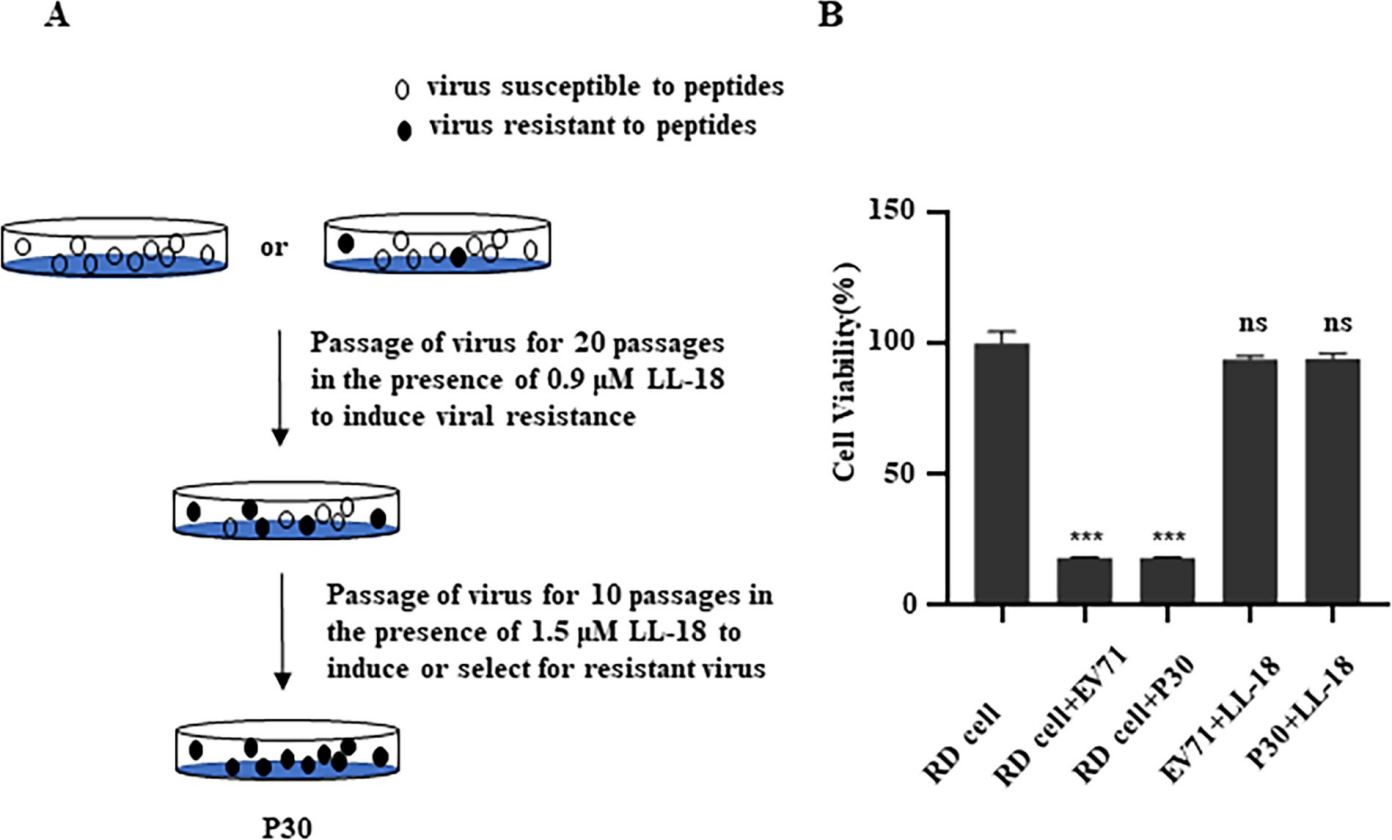
LL-18 did not induce virus resistance. (A) Diagram of resistance induction assay. (B) RD cells infected with parental EV71 or P30 with or without 1.5 μM LL-18 pre-incubation were tested for cell viability 24 h.p.i. ns, not significant; ***, P<0.001.

Viruses, especially RNA viruses, are prone to generate mutations during replication, and thus develop drug resistance [31], posing a great challenge to antiviral development. Selective pressure generated during long-term drug treatment may result in rapid adaptation toward resistance [32]. We therefore tested whether LL-18 is still effective after a long-term treatment. For this purpose, we continuously passaged the virus in the presence of 0.9 μM or 1.5 μM of LL-18 for 30 passages and obtained offspring viruses designated as P30 (Fig 9A). We then tested whether LL-18 was still effective to inhibit P30. Fig 9B showed the P30 viruses were still as susceptible to LL-18 as the parental virus, indicating that peptide therapy is not prone to induce resistant mutants.

## Discussion

EV71 is one of the main pathogens of HFMD and there is an urgent need for effective antivirals against EV71 infection. Small molecules targeting each step of the virus life cycle have been extensively tested and developed in recent years [33]. However, off-target toxicities and the emergence of resistant mutants have posed great concerns and widespread problems for small molecule therapy [34]. On the other hand, therapeutic peptides have higher specificity and efficiency, as well as better safety, making them ideal candidates for pharmacological applications [34, 35]. Here we showed that Cathelicidin peptide analogues were effective against EV71 infection, but could not inhibit Echo virus or coxsackievirus, suggesting these peptides are highly specific. We also determined the susceptibility of the EV71 virus to LL-18 peptide over a longer time of treatment. Our results showed that over 30 passages of successive inhibitory treatment, the viruses were still susceptible to LL-18. Our results suggest that LL-18 binds to a site occupying the interfaces of VP1, VP2, and VP3 interaction. Mutations in this region, together with the conformation changes involved, probably would not affect the VP1, VP2, and VP3 interaction, as dramatic changes in this region will result in virion assembly failure and viral death. That may explain why LL-18 is still effective over a long period.

Both natural and synthetic peptides have been tested for antimicrobial and antiviral purposes. In particular, Cathelicidin peptides have been shown to inhibit a lot of enveloped viruses [14]. When we were preparing this manuscript, Yu et al. showed that human LL-37 could inhibit EV71 infection by regulating antiviral responses [36]. Here we further showed that the two shorter analogues of LL-37, LL-18 and FF-18 showed more potent inhibition against EV71 infection. We also confirmed that LL-18 alone could induce interferon production (S7A Fig). However, pre-incubation of LL-18 with viruses reduced its ability to induce interferon production (S7B Fig). These results indicated that most of the peptides were bound with virions, and therefore, lost their ability to induce interferon production. Yu et al. conducted their experiment in a different way [36], in which they first pre-treated cells with LL-37 and then washed away the peptide. In this scenario, EV71 could still infect host cells as peptide has no direct role on host cells (S3A Fig), explaining why they could still detect interferon production.

LL-18 and FF-18 were obtained by removing the first and last five amino acids of LL-37, which previous studies found to reduce the sensitivity of the peptides to serum inhibition [37], and then replacing the 11^th^ and 20^th^ glutamic and lysine residues with hydrophobic amino acids leucine or phenylalanine, respectively. Previous studies have shown that these modified peptides exhibited robust antimicrobial and antitumor effects [37–39]. Here we further showed that they were also more potent against viral infection, suggesting peptide design/modification is a promising method to identify new antivirals. In particular, in the case of Cathelicidin peptides, increasing the positive charge seems critical to enhance their effects to target negative-charged bacteria, cancer cells [40], or even virions. Alanine substitution of the 11^th^ or 20^th^ residue further showed that the 20^th^ residue is the most important residue mediating its antiviral activity, as LA-18 or AA-18 peptide lost its ability to inhibit viral infection. In addition, lengthy peptides usually have bad bioavailability due to loss of bioactive structure and rapid proteolysis. Therefore, the shorter, 27 aa-long analogues which showed better efficacy in inhibiting viral infection, are superior candidates for future development. We also tried to identify even shorter, functional peptides, but was not successful, suggesting the peptides might require a minimal length to maintain their correct secondary structures for proper function.

Peptides have weak membrane permeability and therefore, most of the active peptides target extracellular molecules [35]. Here we showed that Cathelicidin analogues inhibited EV71 viral entry by directly binding to the virion surface. NMR data showed that LL-18 could interact with the EV71 virion but not the coxsackievirus, explaining why this peptide was specifically effective against the EV71 virus. LL-18 interacts with EV71 virion surface at a site where it could block virus-receptor interaction and uncoating. The binding of SCARB2 to the canyon region is essential to induce conformation changes to initiate the uncoating process. Molecular docking suggested that LL-18 binds to a site adjacent to the canyon and we further showed that this interaction blocks virus-SCARB2 interaction and uncoating.

Another drawback with therapeutic peptides is that they usually have poor *in vivo* stability, and could be easily hydrolyzed or destroyed by enzymes *in vivo* [35]. Due to the lack of suitable and effective animal models, few peptides have been evaluated *in vivo* against EV71 infection. Here, we adapted the EV71 virus strain and established sucking mice infection model with this mEV71 strain. EV71 mouse adaptation has been reported to be associated with mutations in the viral genome, with VP1 145E as the mostly reported critical mutation [28,41]. Notably, the parental EV71 strain that we used for our *in vitro* studies contains 145E in VP1. Sequence analysis of our mEV71 revealed VP1 K98E and 3C P10L mutations and several synonymous mutations in 3C and 3D regions. Interestingly, VP1 K98E has been reported to cooperate with other mutations in VP1 to effectively interact with mouse SCARB2 (mSCARB2) for successful murine cell infection [42]. Therefore, the mEV71 strain here might also be able to utilize mSCARB2 to enhance its mouse tropism. Although with these mutations, we were able to show that LL-18 could still inhibit mEV71 infection *in vitro* and alleviate the detrimental effect of EV71 *in vivo*, suggesting this peptide could be a promising antiviral candidate for further development.

In summary, we here found that two Cathelicidin analogues have excellent anti-EV71 activity both *in vivo* and *in vitro*. Mechanism analysis revealed that these peptides inhibited the binding of the virus to host receptors and viral uncoating processes, thereby preventing infection of host cells. This study provides promising candidates for the development of novel antiviral agents.

## Materials and methods

### Ethics statement

All animal experiments were conducted under protocols approved by the Committee on the Ethics of Animal Experiments of Xi’an Jiaotong University. Specific-pathogen-free (SPF) ICR mice were used for the viral infection model. All institutional guidelines for animal care and use were strictly followed.

### Cells and virus strains

293T cells and human rhabdomyosarcoma (RD) cells were grown in modified Dulbecco Eagle medium (DMEM, GIBCO) containing 10% fetal bovine serum (FBS, BI), 100 U/mL penicillin, 1 μg/mL streptomycin (Beyotime Biotechnology, China), and cultured at 37°C and 5% CO_2_ in a humid incubator.

The EV71 virus (GenBank accession NO. OR800939), Echo7 virus (GenBank accession NO. OR786176), and CVB5 virus (GenBank accession NO. OQ919261) were obtained from Xi’an Municipal CDC, propagated on RD cells for 5 passages for high titers and titrated on RD cells. The NanoLuc-EV71 reporter virus and subgenomic replicon (SGR) have been reported previously [17]. Briefly, the full-length NanoLuc-EV71 reporter virus was propagated on RD cells and the supernatants were harvested, filtered, and stored at -80°C before use. The SGR was *in vitro* transcribed and transfected into cells with TransIT mRNA transfection kit (Mirus Bio, Madison, WI) according to the manufacturer’s instruction.

### Peptides and *in vitro* antiviral tests

Synthetic peptides were purchased from Bioyears Co. Ltd. (Wuhan, China) with purity of more than 95% and dissolved in PBS. To test their inhibitory effects, the EV71 virus (MOI = 1) was incubated with the indicated amount of peptide at room temperature for 1 hr before the mixture was added to RD cells and maintained throughout the experiment. Cell viability, viral titer, or viral protein expression was determined 24 h.p.i. For the time-of-addition experiment, viruses were either pre-incubated with peptides (for the -2 and -1 time points), the mixture was added to the cells simultaneously (for 0 time point), or peptides were added after virus infection (for 1, 2, 4 and 7 h time points) and the virus/peptide was maintained in the culture before cell viability, viral titer, or viral protein expression was determined 24 h.p.i. The TCID_50_ of the virus was calculated according to the Reed-Muench method [43].

### Immunoblotting

Cells were treated as described and harvested with LDS sample buffer (Thermo Fisher Scientific). After denaturation, samples were separated on 10% SDS-PAGE gel and then transferred to a polyvinylidene fluoride (PVDF) membrane. The proteins were blocked using 5% skimmed milk and probed with the corresponding antibodies. The following antibodies were used in this study: EV71 VP1 polyclonal antibody (GeneTex, Taiwan, China), β-Actin antibody (Zen BioScience, Chengdu, China), pan-Enterovirus Monoclonal antibody (Thermo Fisher Scientific). The chemiluminescence signals were visualized using ECL Advance reagents (Mishubio, China), and the images were acquired using the FUSION Solo S Imaging System (VILBER, France).

### Luciferase assay

Full-length NanoLuc-EV71 reporter viruses and subgenomic EV71 replicon (SGR) were described previously [17]. Reporter viruses were preincubated with peptides for 1 hr before infecting RD cells, while RD cells were first transfected with SGR RNA with TransIT-mRNA transfection reagent (Mirus Bio) followed by peptides treatment for 3 or 6 hrs. Nano luciferase and *Renilla* luciferase were measured with Nano-Glo or *Renilla* luciferase assay system from Promega (Madison, WI). Luminescence signals were measured using a BioTek Neo2 microplate reader.

### Cell viability assays

Cell viability assays were carried out using the Cell Titer-Glo Luminescent Cell Viability Assay Kit (Promega, Madison, WI) following the manufacturer’s instructions. Luminescence signals were measured using a BioTek Neo2 microplate reader.

For crystal violet staining, cells treated as indicated were first washed once with PBS, followed by fixation with 4% PFA. Cells were then stained with 0.5% crystal violet for 2 hrs before they were extensively washed with water.

### Virus attachment and internalization assays and Real-time PCR

Viral binding and internalization assays were performed as previously described [44]. Briefly, EV71 viruses were pre-incubated with PBS, 12s, or peptides for 1 hr, before they were used to treat cells (MOI = 1) at 4°C for 1 hr (attachment assay) or incubated at 4°C for 1 hr followed by 1 hr of incubation at 37°C (internalization assay). Cells were then washed extensively with ice-cold PBS, and the viruses retained were analyzed with real-time PCR.

For real-time PCR, cells were treated as described, and cellular total RNA was isolated using the Cell Total RNA Isolation Kit (ForeGene, China) according to the manufacturer’s protocol. The cDNA was synthesized using the ProtoScript II First Strand cDNA Synthesis Kit (New England Biolabs). cDNA was then determined with 2×RealStar Green Fast Mixture (GenStar, China) with the following primers:

β-actin forward: CTGCCGTTTTGCGTAGGAC; β-actin reverse: AGGCGTA CAGGGATAGCAC. VP1 forward: TTGTTACCATATAGCTATTGGATTGGCC; VP1 reverse: CATGTTTAGCTGTGTTAAGGGTCAAGAT. IFN-β forward: GCTTGGATTCCTACAAAGAAGCA; IFN-β reverse: ATAGATGGTCAATGCGGCGTC.

### Nuclear Magnetic Resonance (NMR)

The NMR titrations were performed on a Bruker 800 MHz (Avance NEO) spectrometer equipped with cryo-probe. The molar ratio of the peptide to the virus was 90:1. The experiments were recorded using zgespg pulse for 128 scans at 298k and the results were analyzed by Topspin.

### Co-Immunoprecipitation (Co-IP)

Coimmunoprecipitation was performed as previously described [44]. Briefly, VP1, VP2, and VP3 were PCR amplified and cloned into pcDNA3.1 with an HA tag. LL-18 or LL-37 encoding oligonucleotides were inserted into an eGFP-expressing construct (pHW200-eGFP). SCARB2 and Annexin 2 were PCR amplified from the cDNA library and cloned into pcDNA3.1 with a Flag tag. All constructs were confirmed by sequencing. 293T cells were transfected with the indicated plasmids (500 ng/well) using a FuGENE HD transfection reagent (Promega, Madision, WI) according to the manufacturer’s instructions. 36 hrs later, cells were harvested with IP buffer (20 mM Tris–HCl, pH 8.0, 137 mM NaCl, 1% Nonidet P-40 (NP-40), 2 mM EDTA) supplemented with a protease inhibitor cocktail. After removing cell debris, the supernatant was incubated with HA-antibody or Flag-antibody (OriGene, Rockville, MD) at 4°C for 2 hrs before they were immunoprecipitated with Dynabeads Protein A/G (Thermo Fisher Scientific) and incubated for another 1 hr at 4°C. After washing with wash buffer (0.05% Triton X-100) six times, samples were analyzed by Western blotting.

### Molecular docking

The crystal structure of EV71 was derived from PDB (ID: 4AED), and the secondary structure of peptides was predicted using the CABS dock web server (CABS-dock: server for protein-peptide docking)based on amino acid sequence, and their interactions were simulated with default parameters set by the server.

### Thermal stability assay

EV71 viruses were preincubated with either PBS, NaCl or the indicated amount of LL-18 at room temperature for 2 hrs, before Sybr green II dye (3× working concentration, Solarbio, China) and RNase inhibitor (1 U/μL, Thermo Scientific) were added. The mixture was heated from 25°C to 80°C with an increment of 0.5°C for 30 seconds, and the fluorescence from SYBR green was monitored with a QuantGene 9600 real-time PCR system (Bioer, China).

### Viral particle ultracentrifugation

EV71 viruses were pre-incubated with either PBS, 12s, or LL-18 for 1 hr. Then half the samples were treated with 1.5 μg purified SCARB2 protein (Sino Biological, China) with pH adjusted to 5.5. The samples were incubated at 37°C for 2 hrs to simulate the virus uncoating environment. Viral particles were then loaded onto iodoxanol gradients (0% 500 μL, 21% 1 mL, 27% 1 mL, 33% 1 mL, 39% 1 mL) and ultra-centrifuged at 4°C, 45000 rpm for 1.5 hrs. 7 fractions were collected from the top, and samples were precipitated with Trichloroacetic acid (TCA) before being analyzed with immunoblotting.

### Mice infection

To prepare the mouse-adapted EV71 strain, the parental EV71 virus was intraperitoneally (i.p.) inoculated into 3-day-old ICR mice. Virus was then isolated from the brain tissue and inoculated into RD cells to amplify virus. After four passages in mice, the virus showed enhanced CPE in RD cells and was used as mEV71. 1×10^9^ PFU/ml mEV71 was then incubated with an equal volume of PBS or LL-18 (20 mg/kg) for one hour, and used to infect 6-day-old ICR newborn mice intraperitoneally. Mice were monitored daily to record survival rate. Meanwhile, brain and muscle tissues were collected 1-day post infection for viral titration or H&E staining.

### Induction of resistant virus

Resistance induction assay was conducted as previously reported [45]. Briefly, virus was first successively passaged in RD cells for 20 generations in the presence of a lower concentration of LL-18 (0.9 μM). The progeny virus was then passaged for another 10 generations with 1.5 μM LL-18, and the progeny virus was designated as P30. The parental EV71 and P30 with the same titer were pre-incubated with 1.5 μM LL-18 for 1 h and then tested for cell viability 24 h.p.i.

### Statistical analysis

Unless otherwise indicated, all values represent means ± standard deviations and represent the results of a minimum of three independent experiments. The two-tailed Student’s t-test was used to compare the means of control and experimental groups. ns, not significant; *, P <0.05; **, P<0.01, ***, P<0.001.

## Supporting information

S1 Fig LL-18 and FF-18 effectively inhibit EV71 infection. (A-D) Cell viability of RD cells treated with the indicated amount of DL-37 (A), LL-37 (B), LL-18 (C), or FF-18 (D) was plotted against peptide concentration, and the cytotoxicity was determined. (E) EV71 virus pre-incubated with indicated amounts of DL-37, and the cell viability was determined 24 h.p.i. ***, P<0.001. (F-G) CVB5 (F) or Echo7 (G) virus pre-incubated with 0.3, 0.9, or 1.5 μM of peptides were used to infect RD cells. Cell viability was determined 24 h.p.i. ***, P<0.001. (H-I) CVB5 (H) or Echo7 (I) virus pre-incubated with 3, 6, or 12 μM of peptides were used to infect RD cells. Cell viability was determined 24 h.p.i. ***, P<0.001.
(TIF)

S2 Fig LL-18 and FF-18 inhibit viral attachment and internalization. (A) EV71 virus (MOI = 10) was pre-incubated with 3 μM LL-18 or FF-18 before they were used to incubate with RD cells at 4°C for 1 hr. Cells were then washed extensively and kept cultured for 24 hrs before viral VP1 expression was determined with immunoblotting. (B) EV71 virus (MOI = 10) was pre-incubated with 3 μM LL-18 or FF-18 before they were used to incubate with RD cells at 4°C for 1 hr followed by 37°C incubation for 1 hr. Cells were then washed extensively and kept cultured for 24 hrs before viral VP1 expression was determined with immunoblotting.
(TIF)

S3 Fig LL-18 binds directly to virus particles. (A) RD cells were pre-incubated with indicated amounts of LL-18, FF-18, DL-37, or LL-37 for 2 hrs before they were washed extensively to remove unbound peptides. Cells were then infected with the EV71 virus (MOI = 1) and cell viability was determined 24 h.p.i. Values were normalized to uninfected RD cells. ***, P<0.001. (B). ^1^H NMR spectra of FF-18 peptide in the absence or presence of EV71 virus (green line) or CVB5 virus (red line).
(TIF)

S4 Fig Compound 12s interferes with virus-SCARB2 interaction. 293T cells expressing SCARB2-Flag were lysed and cell lysates were incubated with EV71 virus in the absence or presence of the indicated amount of **12s**. Cell lysates were then immunoprecipitated with anti-Flag antibody and the precipitated virus was determined with anti-VP1 antibody.
(TIF)

S5 Fig LL-18 inhibits mEV71 infection *in vivo*. (A) 6-day-old ICR mice were infected with parental EV71 or mouse-adapted virus (mEV71) and the mice survival rate was determined. (B) RD cells infected with mEV71 pre-incubated with PBS or LL-18 for 1 hr were determined for virus titer 24 h.p.i. ***, P<0.001. (C) ICR mice infected with mEV71 or mEV71 pre-incubated with the indicated amount of LL-18 were sacrificed at 1 d.p.i. and viral titers from muscle were determined. ns, not significant; ***, P<0.001.
(TIF)

S6 Fig Molecular Docking of peptides with EV71. (A-B) Molecular docking of LL1-15 (A) and LL7-21 (B) with EV71.
(TIF)

S7 Fig Induction of IFN-β production by peptides. (A) RD cells treated with 1.5 μM LL-18 or LL-37 for indicated time and IFN-β production was detected by q-RT-PCR. (B) RD cells treated with EV71 viruses pre-treated with PBS or LL-18 for indicated time and IFN-β production was detected by q-RT-PCR. ns, not significant; **, P<0.01; ***, P<0.001.
(TIF)

S1 Table Prediction of interaction sites between peptides and viruses. Viral protein residues highlighted in red interact with SCARB2.
(XLSX)

## References

[1] Patterns of polymorphism and divergence in the VP1 gene of enterovirus 71 circulating in the Asia-Pacific region between 1994 and 2013. Journal of Virological Methods. 2013;193(2):713–28. doi: 10.1016/j.jviromet.2013.07.051 23933074

[2] Solomon T, Lewthwaite P, Perera D, Cardosa MJ, McMinn P, Ooi MH. Virology, epidemiology, pathogenesis, and control of enterovirus 71. Lancet Infect Dis. 2010;10(11):778–90. Epub 2010/10/22. doi: 10.1016/S1473-3099(10)70194-8 . 20961813

[3] Wang H, Li Y. Recent Progress on Functional Genomics Research of Enterovirus 71. Virol Sin. 2019;34(1):9–21. Epub 2018/12/16. doi: 10.1007/s12250-018-0071-9 ; PubMed Central PMCID: PMC6420560. 30552635 PMC6420560

[4] Yuan JJ, Shen L, Wu J, Zou XR, Gu JQ, Chen JG, et al. Enterovirus A71 Proteins: Structure and Function. Frontiers in microbiology. 2018;9. ARTN 286 doi: 10.3389/fmicb.2018.00286 WOS:000425624100002. 29515559 PMC5826392

[5] Plevka P, Perera R, Cardosa J, Kuhn RJ, Rossmann MG. Crystal Structure of Human Enterovirus 71. Science. 2012;336(6086):1274–. doi: 10.1126/science.1218713 WOS:000304905300037. 22383808 PMC3448362

[6] Kobayashi K, Koike S. Cellular receptors for enterovirus A71. Journal of Biomedical Science. 2020;27(1). ARTN 23 doi: 10.1186/s12929-020-0615-9 WOS:000513548800001. 31924205 PMC6954530

[7] Hu KH, Diarimalala RO, Yao CG, Li HL, Wei YH. EV-A71 Mechanism of Entry: Receptors/Co-Receptors, Related Pathways and Inhibitors. Viruses-Basel. 2023;15(3). ARTN 785 doi: 10.3390/v15030785 WOS:000959765900001. 36992493 PMC10051052

[8] Liu Y, Sheng J, Fokine A, Meng G, Shin WH, Long F, et al. Structure and inhibition of EV-D68, a virus that causes respiratory illness in children. Science. 2015;347(6217):71–4. doi: 10.1126/science.1261962 WOS:000347102300050. 25554786 PMC4307789

[9] Smyth M, Pettitt T, Symonds A, Martin J. Identification of the pocket factors in a picornavirus. Archives of Virology. 2003;148(6):1225–33. doi: 10.1007/s00705-002-0974-4 WOS:000183722500016. 12756627

[10] Wang XX, Peng W, Ren JS, Hu ZY, Xu JW, Lou ZY, et al. A sensor-adaptor mechanism for enterovirus uncoating from structures of EV71. Nat Struct Mol Biol. 2012;19(4):424–9. doi: 10.1038/nsmb.2255 WOS:000302514400010. 22388738 PMC3378640

[11] Rossmann MG, He YN, Kuhn RJ. Picornavirus-receptor interactions. Trends in Microbiology. 2002;10(7):324–31. Pii S0966-842x(02)02383-1 doi: 10.1016/s0966-842x(02)02383-1 WOS:000176847300010. 12110211

[12] Huan YC, Kong Q, Mou HJ, Yi HX. Antimicrobial Peptides: Classification, Design, Application and Research Progress in Multiple Fields. Frontiers in microbiology. 2020;11. ARTN 582779 doi: 10.3389/fmicb.2020.582779 WOS:000585623900001. 33178164 PMC7596191

[13] Sorensen OE, Follin P, Johnsen AH, Calafat J, Tjabringa GS, Hiemstra PS, et al. Human cathelicidin, hCAP-18, is processed to the antimicrobial peptide LL-37 by extracellular cleavage with proteinase 3. Blood. 2001;97(12):3951–9. doi: 10.1182/blood.v97.12.3951 WOS:000169164300042. 11389039

[14] Findlay EG, Currie SM, Davidson DJ. Cationic Host Defence Peptides: Potential as Antiviral Therapeutics. Biodrugs. 2013;27(5):479–93. doi: 10.1007/s40259-013-0039-0 WOS:000324490900006. 23649937 PMC3775153

[15] Alagarasu K, Patil PS, Shil P, Seervi M, Kakade MB, Tillu H, et al. In-vitro effect of human cathelicidin antimicrobial peptide LL-37 on dengue virus type 2. Peptides. 2017;92:23–30. doi: 10.1016/j.peptides.2017.04.002 WOS:000402214100004. 28400226

[16] Isogai E, Isogai H, Matuo K, Hirose K, Kowashi Y, Okumuara K, et al. Sensitivity of genera Porphyromonas and Prevotella to the bactericidal action of C-terminal domain of human CAP18 and its analogues. Oral Microbiol Immun. 2003;18(5):329–32. doi: 10.1034/j.1399-302X.2003.00083.x WOS:000185088400012. 12930528

[17] Yang H, Zhao X, Xun M, Ma C, Wang H. Reverse Genetic Approaches for the Generation of Full Length and Subgenomic Replicon of EV71 Virus. Frontiers in microbiology. 2021;12:665879. Epub 2021/06/08. doi: 10.3389/fmicb.2021.665879 ; PubMed Central PMCID: PMC8172962. 34093481 PMC8172962

[18] Xu Z, Tang Q, Xu T, Cai Y, Lei P, Chen Y, et al. Discovery of aminothiazole derivatives as novel human enterovirus A71 capsid protein inhibitors. Bioorg Chem. 2022;122:105683. Epub 2022/03/13. doi: 10.1016/j.bioorg.2022.105683 . 35278779

[19] Zhou D, Zhao Y, Kotecha A, Fry EE, Kelly JT, Wang X, et al. Unexpected mode of engagement between enterovirus 71 and its receptor SCARB2. Nature microbiology. 2019;4(3):414–9. doi: 10.1038/s41564-018-0319-z . 30531980

[20] Dang MH, Wang XX, Wang Q, Wang YX, Lin JP, Sun Y, et al. Molecular mechanism of SCARB2-mediated attachment and uncoating of EV71. Protein Cell. 2014;5(9):692–703. doi: 10.1007/s13238-014-0087-3 WOS:000342123400006. 24986489 PMC4145081

[21] Yang SL, Chou YT, Wu CN, Ho MS. Annexin II Binds to Capsid Protein VP1 of Enterovirus 71 and Enhances Viral Infectivity. Journal of virology. 2011;85(22):11809–20. doi: 10.1128/JVI.00297-11 WOS:000296422700025. 21900167 PMC3209289

[22] Plevka P, Perera R, Yap ML, Cardosa J, Kuhn RJ, Rossmann MG. Structure of human enterovirus 71 in complex with a capsid-binding inhibitor. P Natl Acad Sci USA. 2013;110(14):5463–7. doi: 10.1073/pnas.1222379110 WOS:000318037800054. 23509286 PMC3619292

[23] Smith TJ, Kremer MJ, Luo M, Vriend G, Arnold E, Kamer G, et al. The Site of Attachment in Human Rhinovirus-14 for Antiviral Agents That Inhibit Uncoating. Science. 1986;233(4770):1286–93. doi: 10.1126/science.3018924 WOS:A1986D943800024. 3018924

[24] Nguyen Y, Jesudhasan PR, Aguilera ER, Pfeiffer JK. Identification and Characterization of a Poliovirus Capsid Mutant with Enhanced Thermal Stability. Journal of virology. 2019;93(6). Epub 2018/12/21. doi: 10.1128/JVI.01510-18 ; PubMed Central PMCID: PMC6401428. 30567995 PMC6401428

[25] Meister S, Prunotto A, Dal Peraro M, Kohn T. Salt Enhances the Thermostability of Enteroviruses by Stabilizing Capsid Protein Interfaces. Journal of virology. 2020;94(11). Epub 2020/03/28. doi: 10.1128/JVI.02176-19 ; PubMed Central PMCID: PMC7269450. 32213614 PMC7269450

[26] Yamayoshi S, Ohka S, Fujii K, Koike S. Functional comparison of SCARB2 and PSGL1 as receptors for enterovirus 71. Journal of Virology. 2013;87(6):3335–47. doi: 10.1128/JVI.02070-12 23302872 PMC3592140

[27] Nishimura Y, Shimojima M, Tano Y, Miyamura T, Wakita T, Shimizu H. Human P-selectin glycoprotein ligand-1 is a functional receptor for enterovirus 71. Nature Medicine. 2009;15(7):794–7. doi: 10.1038/nm.1961 19543284

[28] Wang YF, Chou CT, Lei HY, Liu CC, Wang SM, Yan JJ, et al. A mouse-adapted enterovirus 71 strain causes neurological disease in mice after oral infection. Journal of virology. 2004;78(15):7916–24. doi: 10.1128/JVI.78.15.7916-7924.2004 WOS:000222755600007. 15254164 PMC446098

[29] Otvos L, Wade JD. Current challenges in peptide-based drug discovery. Front Chem. 2014;2. ARTN 62 doi: 10.3389/fchem.2014.00062 WOS:000209678600065. 25152873 PMC4126357

[30] Cathal O’Brien DRF, Conleth Feighery. Peptide length significantly influences in vitro affinity for MHC class II molecules. Immunome research. 2008;4(6). doi: 10.1186/1745-7580-4-6 . 19036163 PMC2640366

[31] Pillay D, Zambon M. Antiviral drug resistance. BMJ. 1998;317(7159):660–2. Epub 1998/09/04. doi: 10.1136/bmj.317.7159.660 ; PubMed Central PMCID: PMC1113839. 9728000 PMC1113839

[32] Irwin KK, Renzette N, Kowalik TF, Jensen JD. Antiviral drug resistance as an adaptive process. Virus Evol. 2016;2(1):vew014. Epub 2017/07/12. doi: 10.1093/ve/vew014 ; PubMed Central PMCID: PMC5499642. 28694997 PMC5499642

[33] Lin JY, Kung YA, Shih SR. Antivirals and vaccines for Enterovirus A71. Journal of Biomedical Science. 2019;26(1). ARTN 65 doi: 10.1186/s12929-019-0560-7 WOS:000483770800001. 31481071 PMC6720414

[34] Lalani S, Gew LT, Poh CL. Antiviral peptides against Enterovirus A71 causing hand, foot and mouth disease. Peptides. 2021;136. ARTN 170443 doi: 10.1016/j.peptides.2020.170443 WOS:000614690400008. 33171280 PMC7648656

[35] Wang L, Wang NX, Zhang WP, Cheng XR, Yan ZB, Shao G, et al. Therapeutic peptides: current applications and future directions. Signal Transduct Tar. 2022;7(1). ARTN 48 doi: 10.1038/s41392-022-00904-4 WOS:000755124600001. 35165272 PMC8844085

[36] Yu J, Dai Y, Fu YX, Wang KZ, Yang Y, Li M, et al. Cathelicidin antimicrobial peptides suppress EV71 infection via regulating antiviral response and inhibiting viral binding. Antivir Res. 2021;187. ARTN 105021 doi: 10.1016/j.antiviral.2021.105021 WOS:000657777900004. 33508330

[37] Okumura K, Itoh A, Isogai E, Hirose K, Hosokawa Y, Abiko Y, et al. C-terminal domain of human CAP18 antimicrobial peptide induces apoptosis in oral squamous cell carcinoma SAS-H1 cells. Cancer Lett. 2004;212(2):185–94. Epub 2004/07/29. doi: 10.1016/j.canlet.2004.04.006 . 15279899

[38] Isogai E, Isogai H, Matuo K, Hirose K, Hirata M. Sensitivity of genera Porphyromonas and Prevotella to the bactericidal action of C-terminal domain of human CAP18 and its analogues. Molecular Oral Microbiology. 2010;18(5):329–32. 10.1034/j.1399-302x.2003.00083.x 12930528

[39] Kuroda K, Fukuda T, Yoneyama H, Katayama M, Isogai H, Okumura K, et al. Anti-proliferative effect of an analogue of the LL-37 peptide in the colon cancer derived cell line HCT116 p53+/+ and p53. Oncol Rep. 2012;28(3):829–34. Epub 2012/06/28. doi: 10.3892/or.2012.1876 . 22736062

[40] Kuroda K, Okumura K, Isogai H, Isogai E. The human cathelicidin antimicrobial peptide LL-37 and mimics are potential anticancer drugs. Front Oncol. 2015;5. ARTN 144 doi: 10.3389/fonc.2015.00144 WOS:000359148400001. 26175965 PMC4485164

[41] Zaini Z, McMinn P. A single mutation in capsid protein VP1 (Q145E) of a genogroup C4 strain of human enterovirus 71 generates a mouse-virulent phenotype. J Gen Virol. 2012;93(Pt 9):1935–40. Epub 2012/06/01. doi: 10.1099/vir.0.043893-0 . 22647370

[42] Victorio CB, Xu Y, Ng Q, Meng T, Chow VT, Chua KB. Cooperative effect of the VP1 amino acids 98E, 145A and 169F in the productive infection of mouse cell lines by enterovirus 71 (BS strain). Emerg Microbes Infect. 2016;5(6):e60. Epub 2016/06/23. doi: 10.1038/emi.2016.56 ; PubMed Central PMCID: PMC4932649. 27329847 PMC4932649

[43] Reed LJ, Muench H. A SIMPLE METHOD OF ESTIMATING FIFTY PER CENT ENDPOINTS. American Journal of Epidemiology. 1938.

[44] Zhao X, Yuan H, Yang H, Liu Y, Xun M, Li X, et al. N-Acetyltransferase 8 Promotes Viral Replication by Increasing the Stability of Enterovirus 71 Nonstructural Proteins. Journal of virology. 2022:jvi0011922. Epub 2022/02/17. doi: 10.1128/jvi.00119-22 . 35170979 PMC8941898

[45] Shih SR, Tsai MC, Tseng SN, Won KF, Shia KS, Li WT, et al. Mutation in enterovirus 71 capsid protein VP1 confers resistance to the inhibitory effects of pyridyl imidazolidinone. Antimicrob Agents Chemother. 2004;48(9):3523–9. Epub 2004/08/26. doi: 10.1128/AAC.48.9.3523-3529.2004 ; PubMed Central PMCID: PMC514779. 15328120 PMC514779

