# Supplementary figures and images for: Cathelicidin peptide analogues inhibit EV71 infection through blocking viral entry and uncoating
Source: PLoS Pathog. 2024 Jan 25;20(1):e1011967. doi: 10.1371/journal.ppat.1011967 (PMC10846744; doi:10.1371/journal.ppat.1011967)

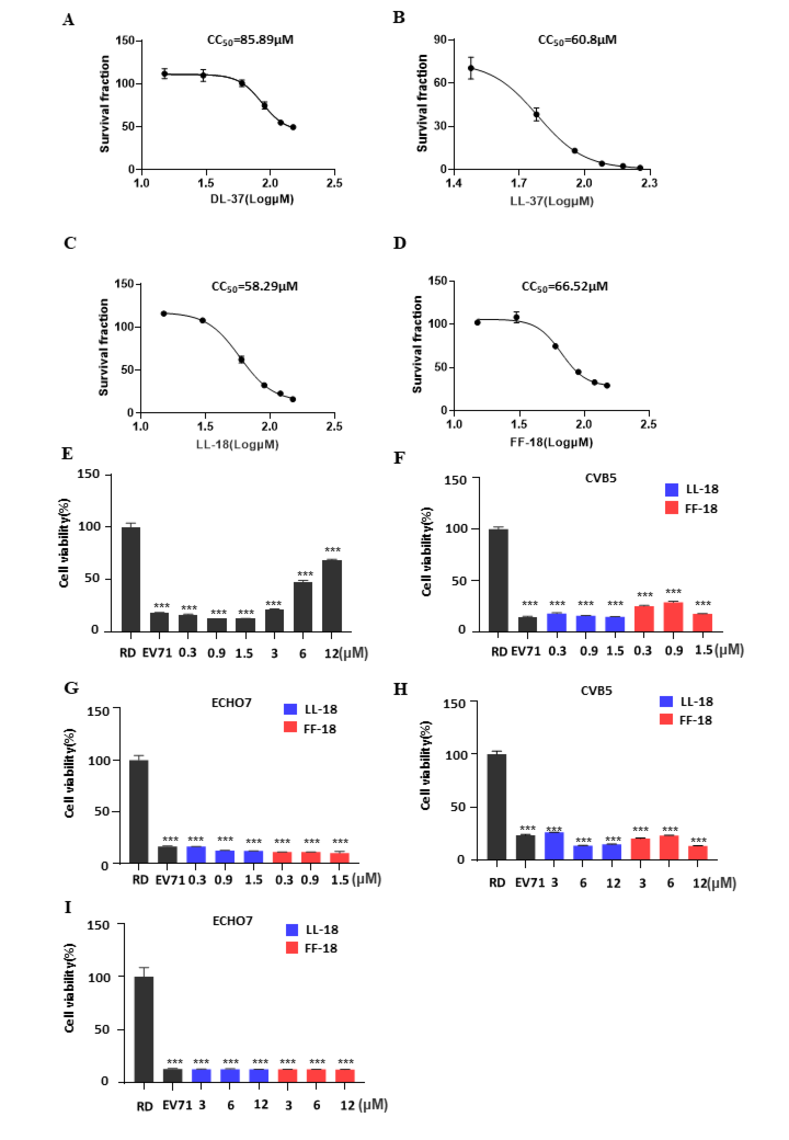

Supplement: S1 Fig — (A-D) Cell viability of RD cells treated with the indicated amount of DL-37 (A), LL-37 (B), LL-18 (C), or FF-18 (D) was plotted against peptide concentration, and the cytotoxicity was determined. (E) EV71 virus pre-incubated with indicated amounts of DL-37, and the cell viability was determined 24 h.p.i. ***, P<0.001. (F-G) CVB5 (F) or Echo7 (G) virus pre-incubated with 0.3, 0.9, or 1.5 μM of peptides were used to infect RD cells. Cell viability was determined 24 h.p.i. ***, P<0.001. (H-I) CVB5 (H) or Echo7 (I) virus pre-incubated with 3, 6, or 12 μM of peptides were used to infect RD cells. Cell viability was determined 24 h.p.i. ***, P<0.001. (TIF) [file ppat.1011967.s001.tif]

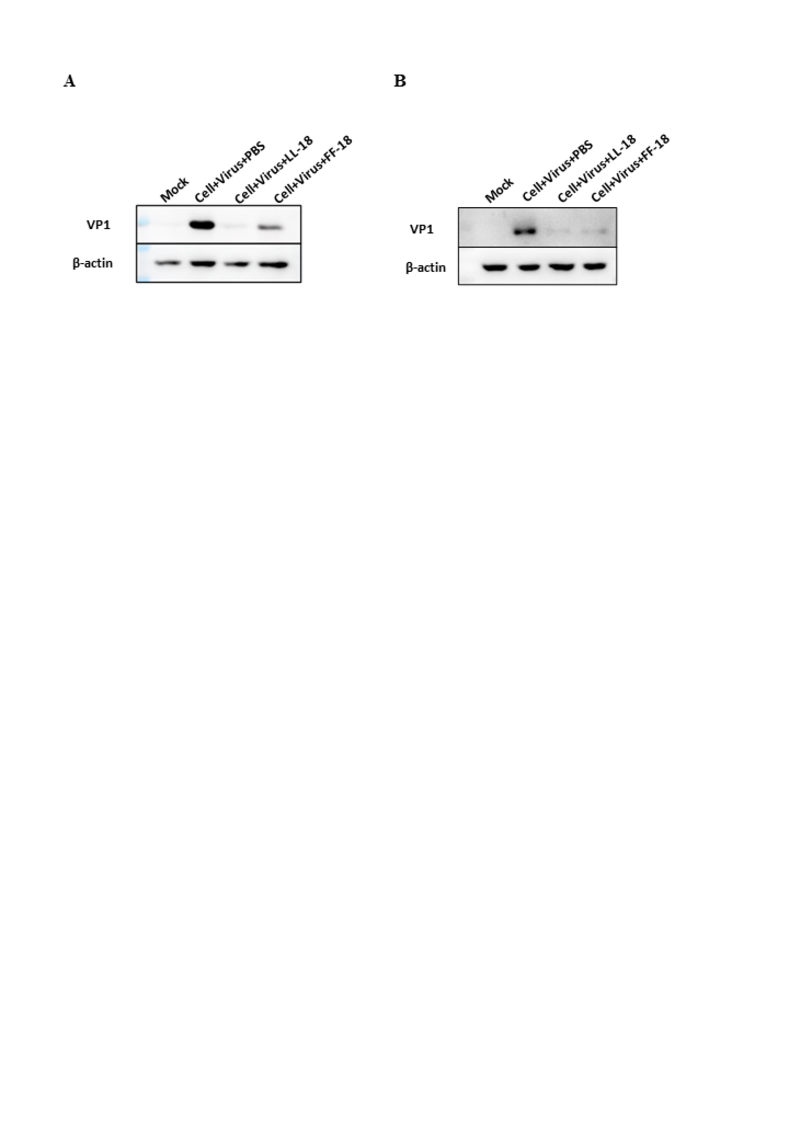

Supplement: S2 Fig — (A) EV71 virus (MOI = 10) was pre-incubated with 3 μM LL-18 or FF-18 before they were used to incubate with RD cells at 4°C for 1 hr. Cells were then washed extensively and kept cultured for 24 hrs before viral VP1 expression was determined with immunoblotting. (B) EV71 virus (MOI = 10) was pre-incubated with 3 μM LL-18 or FF-18 before they were used to incubate with RD cells at 4°C for 1 hr followed by 37°C incubation for 1 hr. Cells were then washed extensively and kept cultured for 24 hrs before viral VP1 expression was determined with immunoblotting. (TIF) [file ppat.1011967.s002.tif]

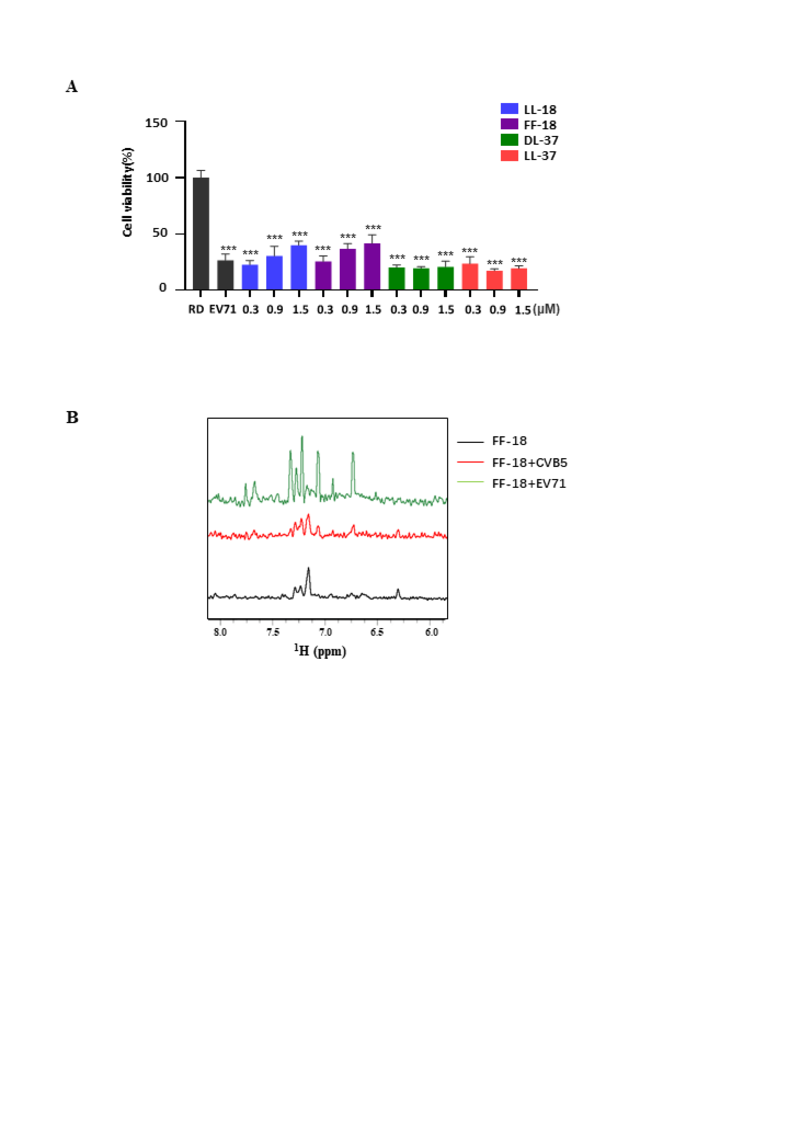

Supplement: S3 Fig — (A) RD cells were pre-incubated with indicated amounts of LL-18, FF-18, DL-37, or LL-37 for 2 hrs before they were washed extensively to remove unbound peptides. Cells were then infected with the EV71 virus (MOI = 1) and cell viability was determined 24 h.p.i. Values were normalized to uninfected RD cells. ***, P<0.001. (B). 1H NMR spectra of FF-18 peptide in the absence or presence of EV71 virus (green line) or CVB5 virus (red line). (TIF) [file ppat.1011967.s003.tif]

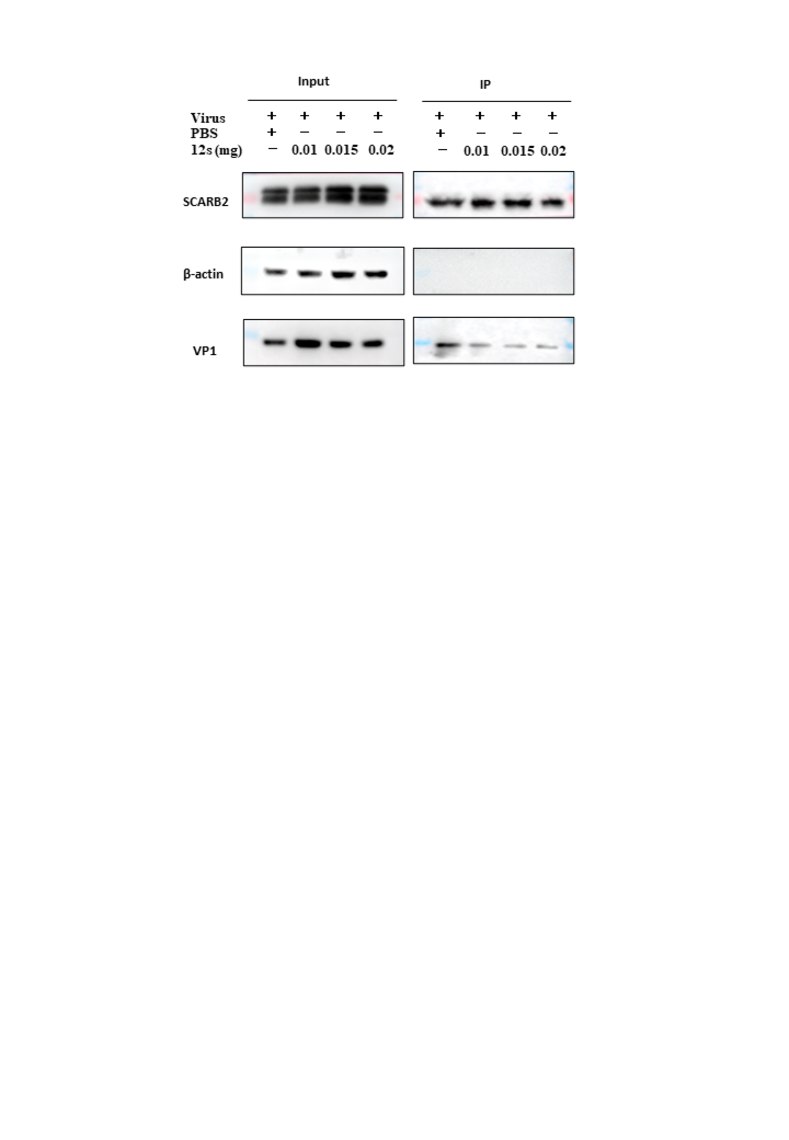

Supplement: S4 Fig — 293T cells expressing SCARB2-Flag were lysed and cell lysates were incubated with EV71 virus in the absence or presence of the indicated amount of 12s. Cell lysates were then immunoprecipitated with anti-Flag antibody and the precipitated virus was determined with anti-VP1 antibody. (TIF) [file ppat.1011967.s004.tif]

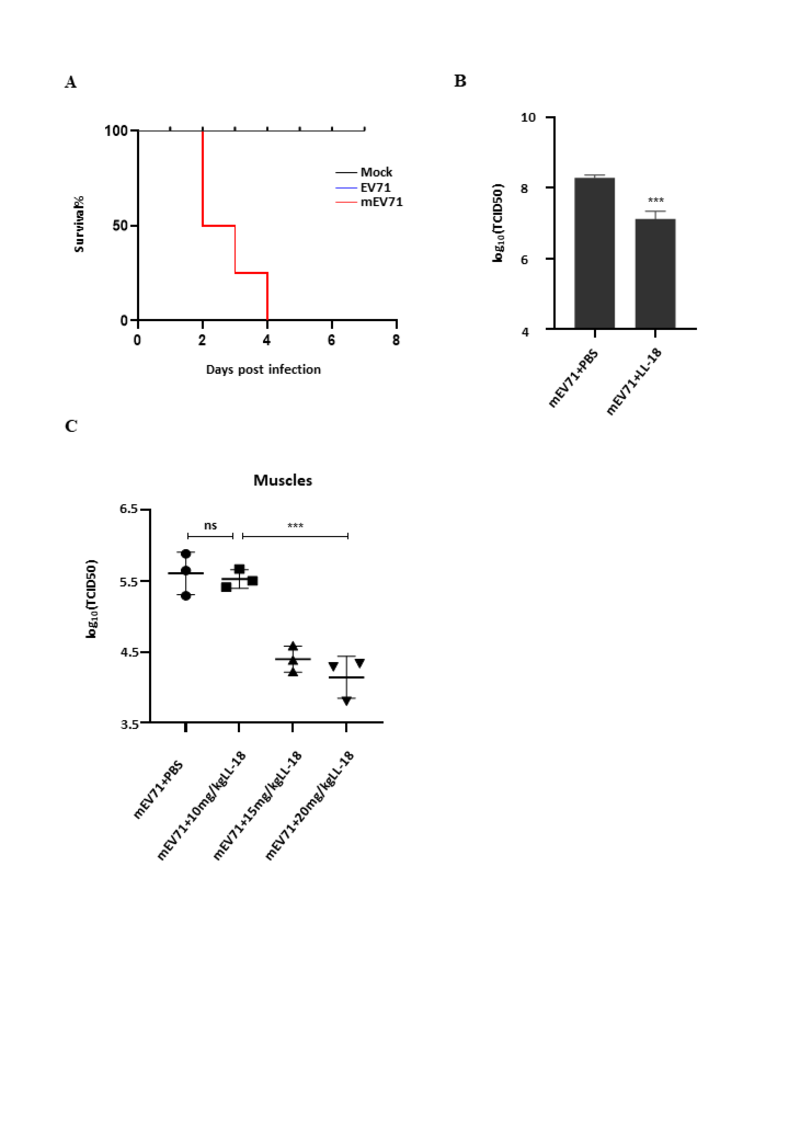

Supplement: S5 Fig — (A) 6-day-old ICR mice were infected with parental EV71 or mouse-adapted virus (mEV71) and the mice survival rate was determined. (B) RD cells infected with mEV71 pre-incubated with PBS or LL-18 for 1 hr were determined for virus titer 24 h.p.i. ***, P<0.001. (C) ICR mice infected with mEV71 or mEV71 pre-incubated with the indicated amount of LL-18 were sacrificed at 1 d.p.i. and viral titers from muscle were determined. ns, not significant; ***, P<0.001. (TIF) [file ppat.1011967.s005.tif]

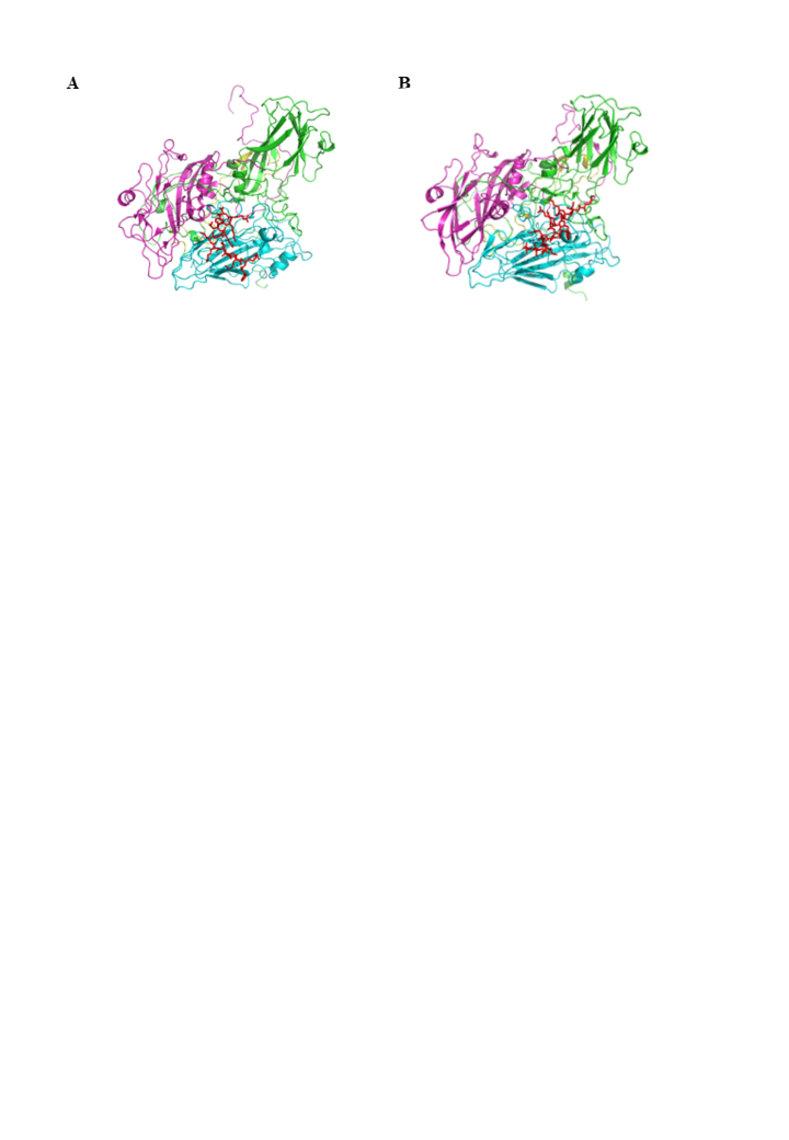

Supplement: S6 Fig — (A-B) Molecular docking of LL1-15 (A) and LL7-21 (B) with EV71. (TIF) [file ppat.1011967.s006.tif]

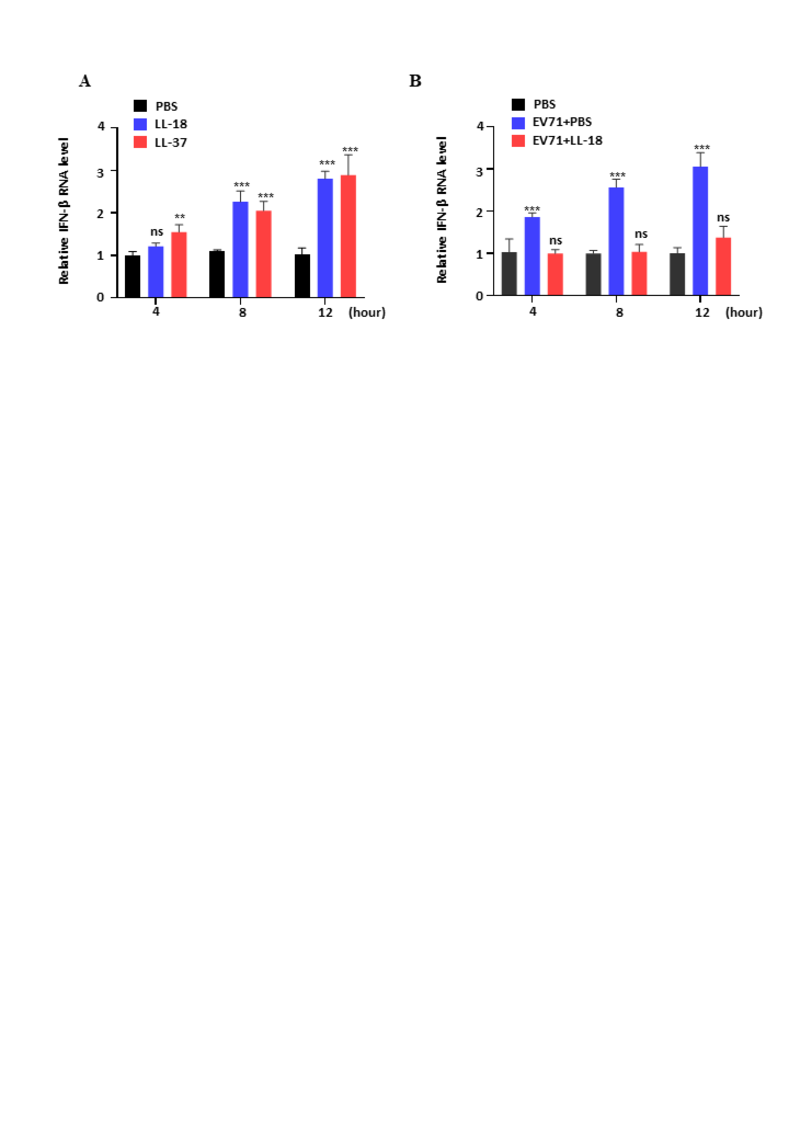

Supplement: S7 Fig — (A) RD cells treated with 1.5 μM LL-18 or LL-37 for indicated time and IFN-β production was detected by q-RT-PCR. (B) RD cells treated with EV71 viruses pre-treated with PBS or LL-18 for indicated time and IFN-β production was detected by q-RT-PCR. ns, not significant; **, P<0.01; ***, P<0.001. (TIF) [file ppat.1011967.s007.tif]
